# Organ-specific apoptotic pathways induced by micro- and nanoplastics: a systematic review of *in vitro* toxicological evidence

**DOI:** 10.3389/ftox.2026.1908508

**Published:** 2026-09-04

**Authors:** Laura Maria Messina, Maria Concetta Scuto, Gabriella Lupo, Cinzia Maria Grazia Lombardo, Caterina Ledda, Angela Trovato Salinaro

**Affiliations:** 1 Department of Biomedical and Biotechnological Sciences, University of Catania, Catania, Italy; 2 Department of Medicine and Surgery, “Kore” University of Enna (UKE), Enna, Italy; 3 Department of Clinical and Experimental Medicine, University of Catania, Catania, Italy

**Keywords:** apoptosis, human health, in vitro toxicology, microplastics, nanoplastics, organ-specific toxicity, oxidative stress

## Abstract

The widespread presence of micro- and nanoplastics (MNPs) in environmental matrices and consumer products has raised increasing concern about their potential effects on human health. Due to their small size, persistence, and heterogeneous physicochemical properties, MNPs may interact with biological barriers, reach different cellular compartments, and trigger organ-specific toxic responses. Among the cellular events involved, apoptosis has emerged as a recurrent mechanism linking MNP exposure to tissue injury and functional impairment. This systematic review aimed to synthesize the available *in vitro* evidence on apoptosis-related toxicity induced by MNPs across different cell models and organoids, with particular attention to organ-specific patterns and underlying molecular pathways. Following a PECOS-based strategy and PRISMA guidance, a literature search was conducted in PubMed, Scopus, and Web of Science on 6 September 2024. Studies were screened using Rayyan, and 80 studies were included in the qualitative synthesis. Overall, the evidence indicates that MNP exposure induces cytotoxic effects in a wide range of cellular models representing the gastrointestinal tract, liver, lung, kidney, reproductive system, placenta, brain, cardiovascular system, skin, and blood-related compartments. Across these models, recurrent mechanisms included oxidative stress, mitochondrial dysfunction, loss of mitochondrial membrane potential, endoplasmic reticulum stress, calcium imbalance, activation of MAPK and PI3K/AKT-related signaling, dysregulation of Bcl-2 family proteins, caspase activation, and crosstalk with inflammatory, autophagic, and ferroptotic pathways. Particle size, surface chemistry, concentration, exposure time, and co-exposure with other contaminants influenced the magnitude and pattern of toxic responses. In conclusion, apoptosis represents a central mechanistic node in the organ-specific toxicity of MNPs *in vitro*. However, the translational relevance of current evidence remains limited by heterogeneity in particle characteristics, exposure conditions, and experimental models. Future studies should prioritize standardized protocols, environmentally relevant exposure scenarios, and advanced human-based models to better define the implications of MNP exposure for human health.

## Graphical Abstract

**Figure:**
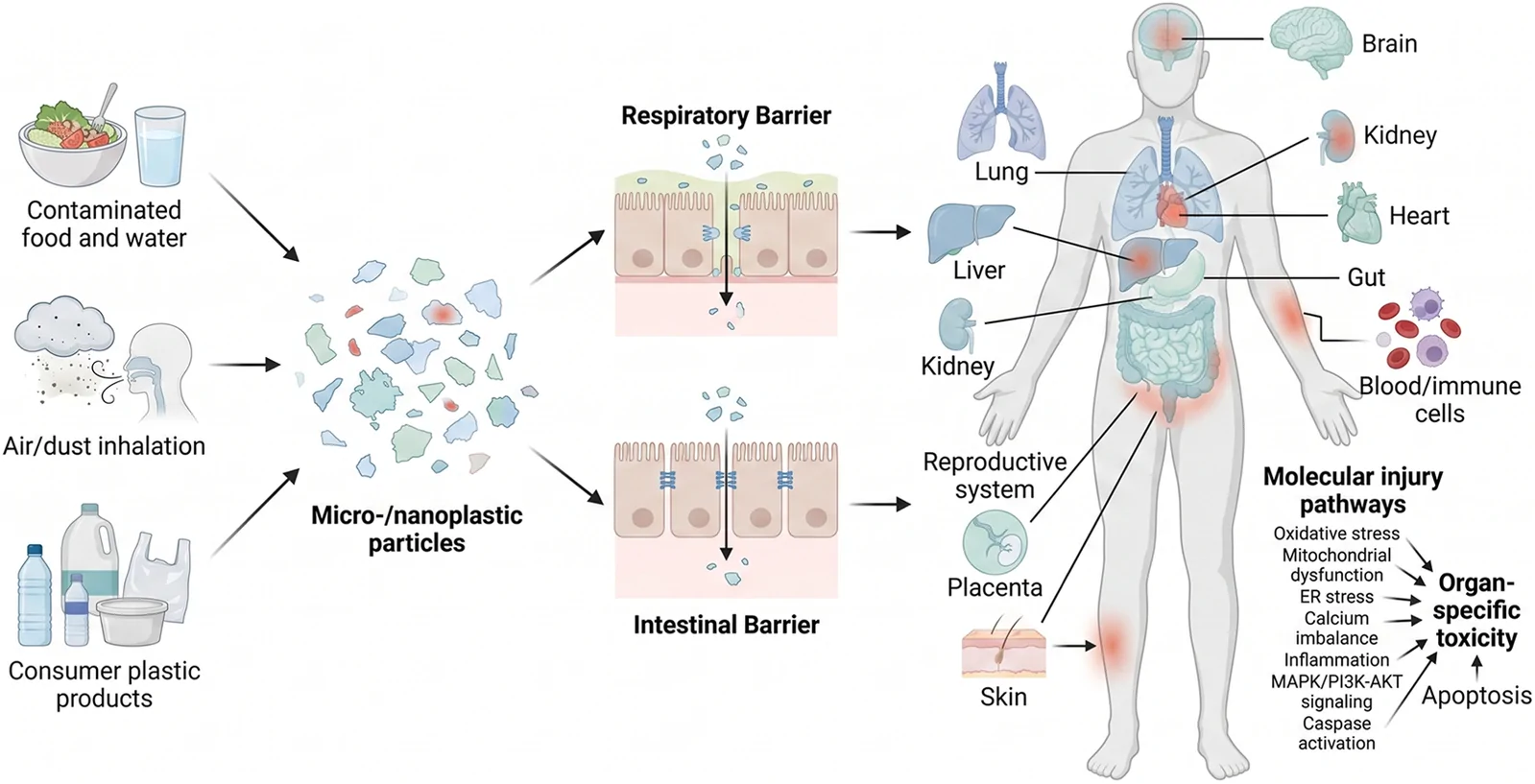

## Introduction

For the purposes of this review, the term micro- and nanoplastics (MNPs) is used as an umbrella concept when the evidence refers to both microplastic and nanoplastic particles. This framing is particularly relevant for the present Research Topic, which focuses on organ-specific toxicity, molecular pathways, and implications for human and environmental health.

Microplastics (MPs), defined as synthetic solid particles or polymers ranging from 1 μm to 5 mm in size, are very widespread environmental contaminants originating from various sources such as household sewage, industrial processes, and the breakdown of larger plastics (Anas and Kunal, 2024; Ghosh et al., 2023; Ziani et al., 2023). These particles appear in multiple forms, including spheres, fragments, and fibers, and are categorized as primary or secondary MPs based on their origin (Yano et al., 2020). Primary MPs are intentionally produced for specific uses, while secondary MPs result from the degradation of larger plastics (Yano et al., 2020).

Nanoplastics (NPs), generally defined as plastic particles smaller than 1 μm, share the same chemical origin as MPs but differ substantially in their biological behaviour. Owing to their smaller size and larger surface-area-to-volume ratio, NPs generally exhibit higher cellular uptake, greater bioavailability, and an enhanced ability to cross biological barriers, including the intestinal epithelium, the blood-brain barrier, and the placental barrier, compared with MPs of the same polymer composition. These properties may translate into distinct organ-specific toxicological profiles. It should be noted that NP-specific *in vitro* toxicological studies are currently less numerous than MP-specific ones in the primary literature; this review reports nanoplastic-specific findings whenever available, and the resulting imbalance in the evidence base is addressed explicitly in the Discussion.

Ubiquitous across marine, freshwater, and terrestrial ecosystems, MPs represent significant risks to living organisms through ingestion, inhalation, and as carriers of other pollutants (Chanda et al., 2024; Li Y. et al., 2024; Stapleton and Hai, 2023). For instance, microplastic ingestion *via* fish consumption has been documented among riverine populations (Labcom et al., 2024), freshwater fish contamination by microplastics represents an emerging exposure pathway (Hayat et al., 2025), and heavy metal pollution in industrial urban soils can further disturb host-associated microbiota (Faurat et al., 2024). Their impact on biological systems is increasingly documented, with significant effects ranging from oxidative stress to tissue damage in marine and terrestrial organisms (Anas and Kunal, 2024; Thacharodi et al., 2024). Alarmingly, MPs have been identified in human biological samples, suggesting potential negative health impacts (Li Y. et al., 2024; Yarahmadi et al., 2024). Addressing the problem of MPs pollution requires advancing detection techniques, promoting sustainable practices, and implementing stronger regulatory frameworks (Albaseer et al., 2024; Matavos-Aramyan, 2024; Nikpay and Toorchi Roodsari, 2024; Pang et al., 2021).

MPs primarily enter the human body through ingestion, inhalation, and, to a lesser extent, dermal contact, with ingestion and inhalation identified as the main routes (Liu and You, 2023; Prata et al., 2020). They have been detected in various human organs and systems, including the digestive, respiratory, and cardiovascular systems (Enyoh et al., 2023; Li Y. et al., 2024; Liang et al., 2024; Persiani et al., 2023). Reported toxic effects include cellular damage, inflammation, and DNA damage, and MPs can also serve as carriers for other pollutants and pathogens (Kadac-Czapska et al., 2024; Pang et al., 2021; Sun N. et al., 2023; Wright and Kelly, 2017; Zhao et al., 2024). Additional evidence indicates that heavy-metal accumulation carried by environmental contaminants can induce oxidative status alterations and histopathological changes across multiple organs (Tanhan et al., 2025). Chronic exposure may lead to accumulation in tissues and organs, potentially causing long-term health issues, particularly affecting the digestive and respiratory systems (Winiarska et al., 2024; Zhu J. et al., 2024).

MPs taken orally are partly excreted through feces (Zhu L. et al., 2024). Several types of MP have been detected in human fecal samples (Hartmann et al., 2024; Schwabl et al., 2019; Zhang et al., 2021), suggesting a role in shaping gut microbiota composition, which contributes to immune maturation and maintenance of epithelial barrier integrity in the environment (Kadry et al., 2021; Lupo et al., 2025). Impairment of this barrier increases permeability to bacterial components and metabolites, and disturbance of microbial balance favors overgrowth of pathogenic bacteria and pathobionts may occur. Several studies suggest intestinal dysbiosis is associated with the onset of various pathological conditions (Lu et al., 2019; Yan et al., 2022).

Although research on the specific health impacts in humans remains limited, the existing evidence highlights the need for further studies on the long-term consequences of MPs exposure (Wu et al., 2022).

Beyond these organ-specific and gut-microbiota-related effects, an overarching cellular mechanism common to many of the pathological outcomes described above is apoptosis. Apoptosis, a programmed form of cell death, plays a vital role in tissue homeostasis, development, and immune function (Elmore, 2007; Mondello and Scovassi, 2009). Recent evidence corroborates the broad relevance of apoptosis across diverse pathological contexts (Xian et al., 2024). Characterized by distinct morphological and biochemical cellular changes such as chromatin condensation and DNA fragmentation (Chen et al., 2024), apoptosis is regulated by complex signaling pathways involving mitochondria, caspases, and Bcl-2 family proteins (Robertson and Orrenius, 2000). These processes are orchestrated through several interconnected signalling cascades, including the intrinsic (mitochondrial) and extrinsic (death receptor) pathways, reactive oxygen species (ROS)-mediated signalling, the caspase cascade, and modulation by MAPK, PI3K/Akt, NF-κB, and p53 pathways, which recur throughout the organ-specific evidence discussed below. In addition, several intracellular signaling pathways, including the JAK-STAT pathway, have been implicated in the regulation of apoptosis by modulating the expression of pro- and anti-apoptotic genes, thereby influencing cell survival and death responses under environmental stress conditions (Wu Z et al., 2024). Environmental toxicants can trigger apoptotic processes, potentially contributing to various diseases, including cancer and neurodegenerative disorders, with oxidative stress often playing a key role in toxicant-induced apoptosis (Franco et al., 2009; Johnson and Sarosiek, 2024; Ledda et al., 2016). Dysregulation of apoptosis—whether excessive or insufficient—can be detrimental to cellular health, underscoring the importance of understanding how environmental exposures, such as MPs, modulate these pathways (Corcoran et al., 1994).

Despite this growing mechanistic insight, it remains unclear whether apoptotic responses to MNP exposure differ systematically across organ systems, and a dedicated organ-specific synthesis of the available *in vitro* evidence has so far been lacking.

This systematic review aimed to examine apoptosis-related mechanisms induced by micro- and nanoplastics and to interpret the available *in vitro* evidence through an organ-specific toxicological lens, with emphasis on convergent molecular pathways and potential implications for human health.

## Materials and methods

### Study design and protocol

This systematic review aimed to elucidate the extent to which MNP exposure may trigger apoptotic mechanisms, negatively influence cellular homeostasis, and increase susceptibility to organ-specific dysfunction. The synthesis focused on apoptosis-related pathways and health implications across different physiological systems and experimental *in vitro* models.

Applying the PECOS framework (Morgan et al., 2018), the review synthesizes the results obtained from different cell lines derived from human tissues, exposure levels, apoptotic mechanisms, and health implications.

This structured PECOS (Morgan et al., 2018; Morgan et al., 2016) approach ensures a rigorous selection process, focusing specifically on apoptotic mechanisms induced by MPs exposure across *in vitro* cell models and supporting a comprehensive synthesis of evidence in the context of human health implications.

The specific PECOS criteria applied to define eligibility are outlined in Table 1.

**TABLE 1:** PECOS criteria for study selection.

Population	All studies that use relevant cell lines or organoids derived from human tissue are eligible. This includes primary cells, immortalized cell lines, and 3D organoid models that mimic human tissue structures
Exposure	The study must involve exposure to micro- and nanoplastics, with a clear description of the concentration, type, and duration of exposure to ensure relevance and comparability
Comparison	Comparison conditions (such as non-exposed control groups or different microplastic concentrations) will be assessed at a later stage, depending on the consistency and variety of data available
Outcome	Studies must report apoptosis-related outcomes, including but not limited to indicators like caspase activity, oxidative stress markers, mitochondrial changes, or DNA fragmentation
Study type	Eligible studies include experimental laboratory studies that report quantifiable apoptosis-related outcomes in response to micro- or nanoplastic exposure

Following the formulation of the review questions, the authors established an *a priori* protocol, as described by Van Den Akker et al. (2023). This systematic review has been conducted in accordance with the PRISMA guidelines, where applicable (Moher et al., 2009; Page et al., 2021), to ensure the transparency and reproducibility of the methodology employed.

A comprehensive search was conducted, on 6 September 2024, in three electronic databases: PubMed, Web of Science and Scopus (Moher et al., 2009). The search strategy on PubMed utilized the following terms: (“Microplastics” [Mesh] OR “Plastic Particles” [Mesh] OR “Environmental Contaminants” [Mesh]) AND “Apoptosis” [Mesh]. The search on Scopus was carried out using the following terms: (TITLE-ABS-KEY (microplastics AND apoptosis) OR TITLE-ABS-KEY (plastic AND exposure AND apoptosis) OR TITLE-ABS-KEY (microplastic AND cellular stress) OR TITLE-ABS-KEY (microplastics AND oxidative stress)) AND TITLE-ABS-KEY (human OR mammalian OR cell OR organoid). The Web of Science search used the following terms: (“microplastics” AND “apoptosis”) OR (“plastic” AND “exposure” AND “apoptosis”) OR (“microplastic” AND “cellular stress”) OR (“microplastics” AND “oxidative stress”) AND (“human” OR “mammalian” OR “cell” OR “organoid”).

In response to peer review, a supplementary search restricted to PubMed was performed on 30 June 2026 using the same search string, to identify literature published between 1 January 2025 and the submission date. Additional relevant studies identified through this update, together with further studies suggested during peer review, have been incorporated into the relevant organ-specific subsections of the Results and Discussion and added to the reference list; a full re-screening of all three databases (PubMed, Web of Science, Scopus) was not repeated in its entirety for this revision (see Limitations).

These strategies were designed to capture relevant studies related to MPs exposure and apoptosis mechanisms, ensuring a thorough collection of data for analysis in this review.

The systematic review is registered on OSF Registries (osf.io/ex5tk).

### Study selection process and eligibility criteria

Following the initial identification of search results, peer-reviewed citations were extracted from major databases. The Rayyan software (2024) was utilized to streamline the removal of duplicate records and facilitate the review and management of the articles selected for inclusion in this systematic review. Three independent reviewers (L.M.M., A.T.S., and C.L.) participated in the selection process.

The screening procedure was conducted in two distinct stages. In the first stage, two independent reviewers (L.M.M. and A.T.S.) separately evaluated the titles, abstracts, and keywords of the identified articles to assess their relevance and suitability for inclusion in the review. Articles deemed unrelated to the scope of this systematic review were excluded. Relevant review articles were considered in this stage for potential citation searching, serving as valuable sources for articles not indexed in the selected databases (Briscoe et al., 2020).

In the secondary screening stage, the same reviewers assessed the full-text manuscripts based on predefined inclusion and exclusion criteria to determine eligible studies. Any discrepancies between the two reviewers were resolved by the third reviewer (C.L.), who oversaw the review process and provided consultation throughout both stages.

Included studies had to meet the following criteria: (1) original experimental research articles, (2) studies investigating the effects of MPs exposure on apoptosis mechanisms or related cellular outcomes, and (3) studies published in English. Exclusion criteria included (1) review articles, case reports, conference papers, and editorials, and (2) studies that lacked sufficient data for meaningful analysis.

Data were extracted by two independent reviewers (L.M.M. and A.T.S.) using a predefined extraction form. Any disagreements between the reviewers were resolved through discussion or consultation with a third reviewer (C.L.).

Data synthesis was conducted qualitatively. Given the heterogeneity of cell models, particle types, exposure metrics, and outcome measures across the included studies, a quantitative meta-analysis was not considered appropriate. Findings were grouped and compared within organ-specific categories (e.g., gastrointestinal, hepatic, pulmonary, renal, reproductive) to identify convergent and divergent apoptosis-related mechanisms, as reported in the Results and Discussion sections.

### Assessment of risk of bias, study quality, and evidence levels

The quality and risk of bias of the included studies were independently evaluated by two reviewers, with all disagreements resolved through discussion with field experts. The assessment of study quality was performed using the BEES-C instrument for human studies and the Toxicological Data Reliability Assessment Tool (ToxRTool) for *in vitro* studies (LaKind et al., 2014).The BEES-C instrument classified articles into three tiers based on relevance: TIER 1 (most relevant), TIER 2 (relevant), and TIER 3 (not so relevant) (LaKind et al., 2014). The ToxRTool, which assigns a maximum score of 21 for *in vivo* studies and 18 for *in vitro* studies, was used to evaluate reliability. Following established guidelines, *in vitro* studies were classified as: useful (15–18), potentially useful (11–14), and not useful (<11 or if any red criteria were unmet) (LaKind et al., 2014; Schneider et al., 2009).

## Results

### Literature search

**FIGURE 1:**
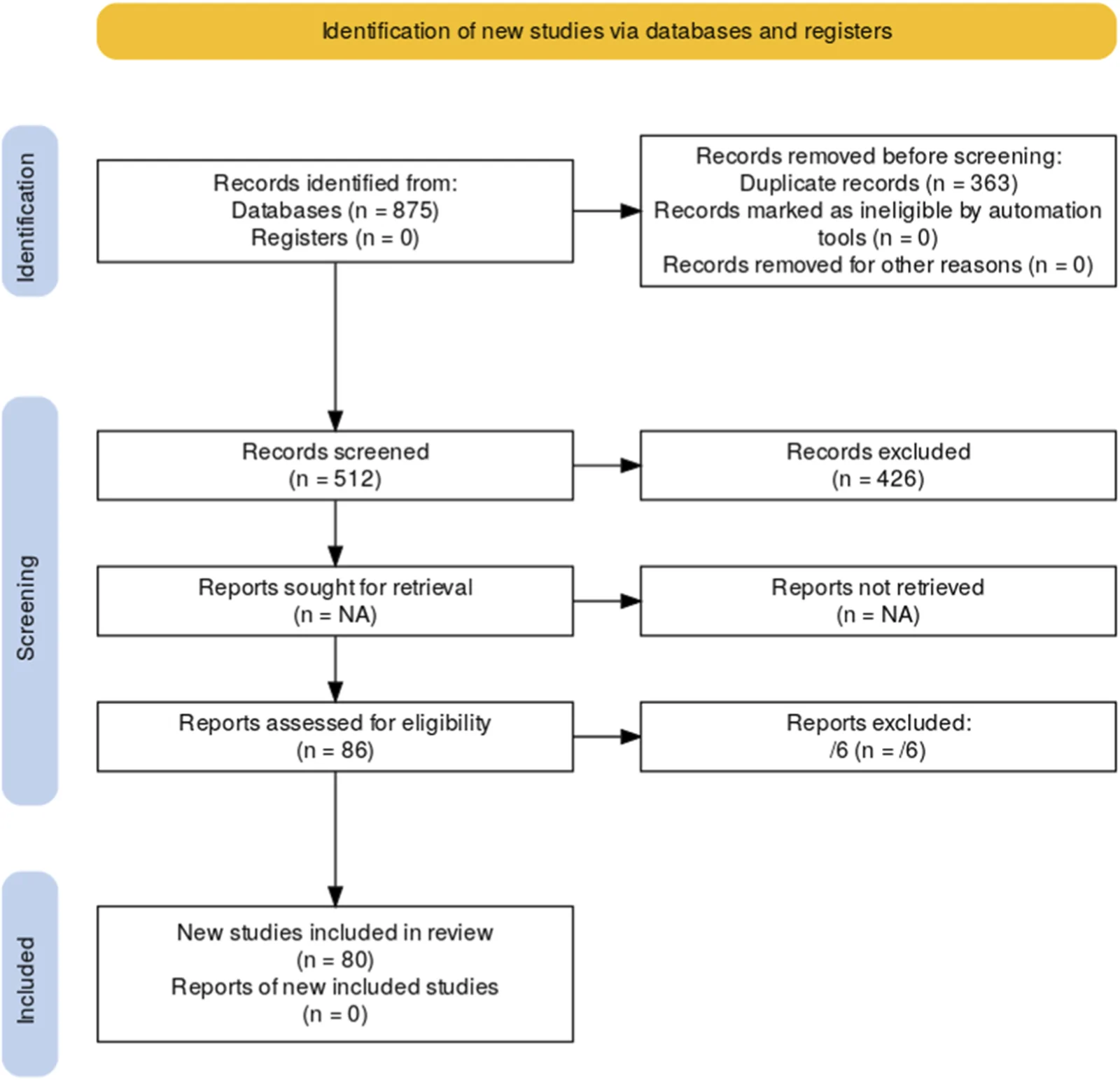
PRISMA diagram for the systematic literature review.

A comprehensive search across multiple databases initially identified 875 records. The PRISMA flowchart delineates the selection process of pertinent publications at each stage of the systematic review (Figure 1). Following the removal of 363 duplicate records, 512 records proceeded to the screening phase. After title/abstract screening, 86 reports were assessed for eligibility, and 80 studies were ultimately incorporated into the qualitative synthesis.

### Characteristics of the included studies

**FIGURE 2:**
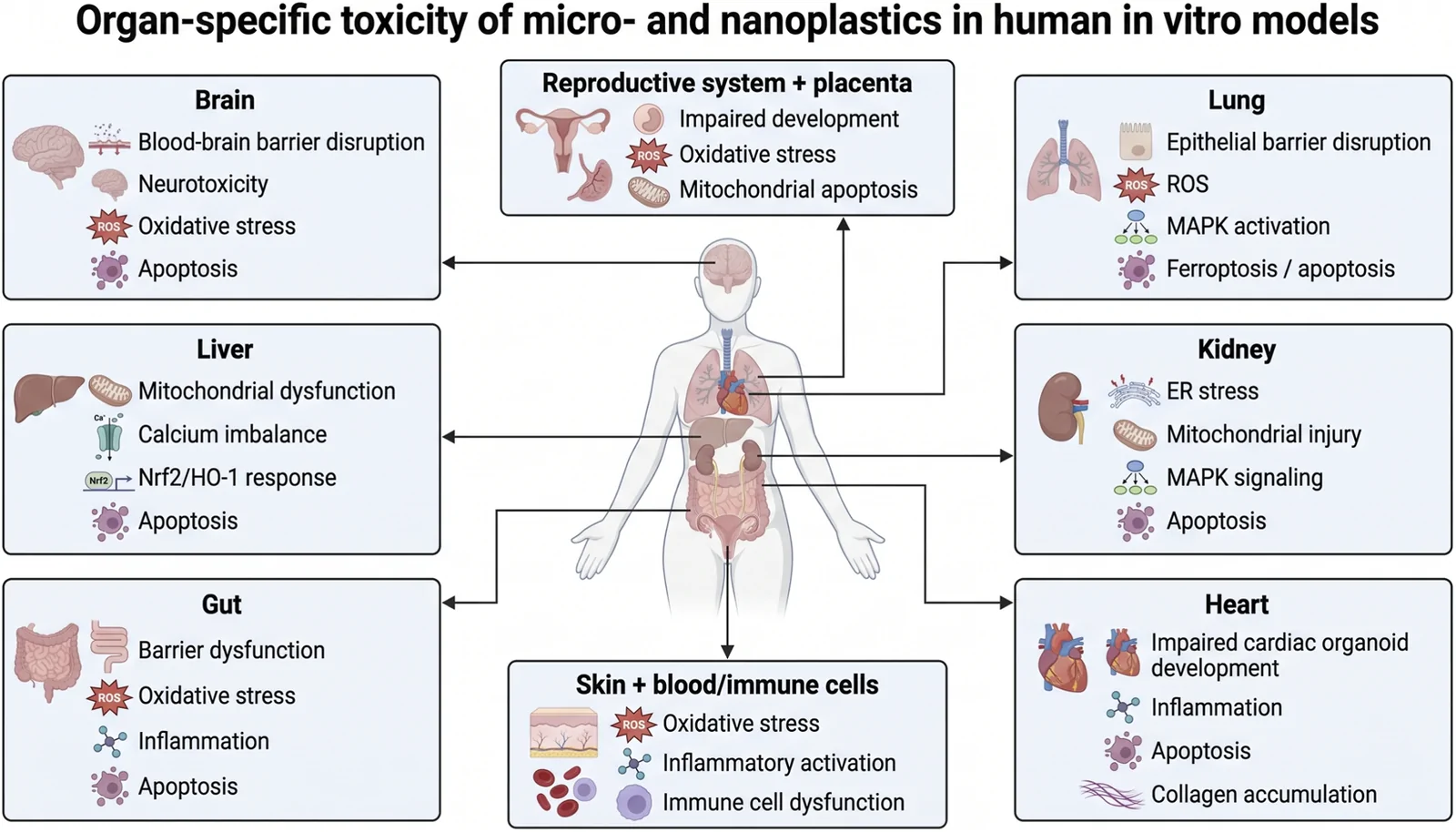
Organ-specific toxicity of micro- and nanoplastics. The figure summarizes the main organ systems and human *in vitro* models affected by micro- and nanoplastic exposure. Across gastrointestinal, hepatic, pulmonary, renal, reproductive, placental, neural, cardiovascular, cutaneous, blood, and immune-related models, recurrent toxicity mechanisms included oxidative stress, mitochondrial dysfunction, endoplasmic reticulum stress, calcium imbalance, inflammation, barrier disruption, and apoptosis-related signaling. These mechanisms support apoptosis as a convergent endpoint of organ-specific micro- and nanoplastic toxicity. Created in BioRender. Ledda, C. (2026) https://BioRender.com/nuhq2uf.

The included studies were classified by organ and cell category using MeSH terms and reported cellular models. As shown in Table 2, cells of various types, especially epithelial cells such as Caco-2, A549, L02, HEK293, HIEC-6, HT-29, HeLa, MCF-7, GES-1, HepaRG, AGS, SH-SY5Y, FLS, Jurkat cells, HUVECs, and others, were used to assess cell viability and apoptosis-related outcomes after exposure to defined concentrations of MNPs, either alone or in combination with food matrices or other pollutants of concern. Epithelial cells were the most commonly represented, with most studies focusing on gut, lung, liver, and kidney models. Other investigated models included stem cells, connective tissue cells, endocrine cells, germ cells, endothelial cells, immune cells, and organoids. This categorization highlights the diversity of biological systems affected by MNP exposure and supports an organ-specific interpretation of apoptosis-related toxicity (Figure 2). It should be noted that the large majority of the included studies investigated microplastics, with comparatively fewer studies specifically addressing nanoplastics; this reflects the current distribution of the primary *in vitro* literature rather than a selection bias of the present review.

**TABLE 2:** Distribution of studies by organ and cell type investigating micro- and nanoplastic exposure and apoptosis-related outcomes.

Organ	References	Cell type	Exposure concentration	Exposure time	Outcome	ToxTool (Max 18)	BEES-C
Gut	Jin et al. (2024)	Caco-2	9:1 mixture of medium: raw leachate/supernatant/resuspended MPs was added	48 h	Oxidative stress, redox imbalance, metabolic dysfunction	17	Tier 1
	Stock et al. (2022)	Caco-2, HT29-MTX-E12	5 × 10^9^; 1 × 10^10^; 2.5 × 10^10^; 5 × 10^10^; 1 × 10^11^; 2.5 × 10^11^; 5 × 10^11^; and 1 × 10^12^	24 h	Particle size- and surface-chemistry-dependent cellular uptake and toxicity	16	Tier 1
	Domenech et al. (2021)	Caco-2	0.26 μg/cm^2^ of y-PSNPs; 0.0006, 0.26, 1.3 and 6.5 μg/cm^2^ for PSNPs	8 weeks	Ultrastructural and molecular alterations	18	Tier 1
	Yan et al. (2023)	Caco-2	PS20/PS1000: 0, 0.1, 10, 50, 100, 500 μg/mL OA: 0, 1, 10, 20, 40, 60, 80 ng/mL	24 h	ER stress, mitochondrial damage, caspase activation, apoptosis (PS-NPs > PS-MPs; potentiated by OA)	17	Tier 1
	Zhang Y et al. (2022)	CCD841CoN HIEC-6	PS-MPs:12.5, 25, 50, and 100 μg/mL	0.5, 1, 4, 8, 12, 24 h	Membrane damage (PS-MPs > PS-NPs); time-dependent PS-NP absorption	18	Tier 1
	K C et al. (2023)	Caco-2	PTFE-MPs (6.0 or 31.7 μm) 1–1,000 μg/mL	24 h	Increased intracellular ROS and nitric oxide production (size-independent)	18	Tier 1
	Ding et al. (2024)	Caco-2	Caco-2: NP 40 μmol/L, 500 μg/mL PS-MPs 500 mg/mL PS-MPs with pre asorbed NP. Caco-2 cell monolayer short time: 40 μmol/L NP, 500 μg/mL PS-MPs and PS-MPs pre-absorbed with NP. Caco-2 cell monolayer long time: 8 μmol/L NP, 100 μg/mL PS-MPs and PS-MPs pressored with NP	6 h; 48 h 6 days	Oxidative stress, inflammation, MAPK-mediated proliferation changes, apoptosis	18	Tier 1
	Li et al. (2023e)	Caco-2	PS-NPs: 10.84, 21.68 μg/mL	72 h	Enhanced PS-NP absorption with food matrices; inflammation, cellular stress, apoptosis	17	Tier 1
	Ding et al. (2022)	Caco-2	40 μmol/L NP, 500 mg/L PS-MPs and 0.1 μm PS-MPs with NP	12, 48 and 72 h	Reduced cell proliferation	18	Tier 1
	Park S. B. et al. (2023)	CCD18-Co cells, colon organoid	CCd18: 0.008, 0.04, 0.2, 1, 5, 10 mg/mL of MPs Colon Organoids: 00,003–5 mg/mL of MPs	12–168 h	Reduced organoid viability; increased inflammation/apoptosis/immunity gene expression	17	Tier 2
	Huang W et al. (2021)	Caco-2	PEMPs: 25,100 and 1,000 mg/L TBBPA: 5, 10, 25 and 50 mg/L	12 h	Reduced Caco-2 cell status (PEMP and TBBPA)	18	Tier 1
	Hou et al. (2022)	Intestinal organoid	PS-NPs: 10, 50, 100, 150, and 200 μg/mL	24 and 48	Cellular accumulation, apoptosis, inflammation; reduced by endocytosis inhibition	17	Tier 1
	Wang et al. (2020)	Caco-2	PS: 20, 50, 70, 90 and 120 μg/mL	24 h	Increased cytotoxicity, oxidative stress, mitochondrial depolarization	18	Tier 1
	Liu et al. (2022)	Caco-2	PS and PFOS: 5, 10, 20, 40 and 80 μg/mL Combined treatment: 10 μg/mL PS and 10 μg/mL PFOS then 10 μg/mL PS and 20 μg/mL PFOS	24 h	Increased intracellular ROS and mitochondrial membrane potential	17	Tier 1
	Busch et al. (2021)	Caco-2, HT29-MTX-E12	PE: 0–50 μg/cm^2^ PE: 0–160 μg/mL	24 h	Cytotoxicity, pro-inflammatory effects	15	Tier 1
Liver	Huang et al. (2023a)	HepaRG cells	5 × 10^9^; 1 × 10^10^; 2,5 × 10^10^; 5 × 10^10^; 1 × 10^11^; 2,5 × 10^11^; 5 × 10^11^; 1 × 10^12^	24 h	Dose-dependent decrease in cell viability; preferential uptake of neutral PS particles	15	Tier 1
	Li et al. (2023d)	Human normal liver cells (LO2 cells)	nPS in particle sizes of 20, 60, 100, 500, and 1,000 nm at concentrations of 1, 5, 25, 75, 125 μg/mL	12 h	Size- and concentration-dependent toxicity; smaller particles increase cell mortality	16	Tier 2
	Stock et al. (2022)	HepaRG cells	5 × 10^9^; 1 × 10^10^; 2,5 × 10^10^; 5 × 10^10^; 1 × 10^11^; 2,5 × 10^11^; 5 × 10^11^; 1 × 10^12^	24 h	Particle size, surface modification and hydrophobicity affect uptake/toxicity	15	Tier 1
	He et al. (2024)	HepG2 cells	PS 20 nm:10 mg/mL PS 50 nm: 100 mg/mL PS 200 nm: 100 mg/mL PS 500 nm: 100 mg/mL	24 h	Reduced mitochondrial membrane potential; enhanced arsenic-induced apoptosis (size-dependent)	15	Tier 1
	Li et al. (2021a)	L02 cells	0.2, 0.4 and 0.6 mg/mL	24 h	Elevated intracellular Ca2+, SOC activation	18	Tier 1
	Pan et al. (2021)	L02 cells	0.5 mg/mL	24 h	Increased CHOP/caspase-12 expression; PERK silencing reduces ROS/p62	16	Tier 1
	Ma et al. (2024)	HepG2	0, 10, 20, 50, 100, and 150 μg/mL	24 h	Mitochondrial apoptotic pathway activation	17	Tier 1
	Li et al. (2023a)	HepG2	0, 6.25, 12.5, 25 and 50 μg/mL	24 h	Mitochondrial injury, reduced ATP, loss of MMP	18	Tier 1
	Li et al. (2021b)	L02	0.5 mg/mL of mic-PS and 100 μM of NaHS	24 h	Reduced inflammation/apoptosis/oxidative stress *via* Nrf2/HO-1/NQO1 activation	18	Tier 1
	Zheng et al. (2023)	L02	BPA, PCP, Pb(NO3)2 at concentrations of 22.3, 48.6, 105.6, 230.0, or 500.0 μM, or PS with concentrations of 53.9, 116.2, 250.0, 538.9, and 1,160.9 μg/mL	48 h	Additive toxicity with BPA, PCP, Pb2+	17	Tier 1
	Wang, L. et al. (2021)	SMMC-7721 cells	2, 5, 10, 20, 50, or 100 μg/mL PS-MPs or PS-MPs+	24 h	Morphological alteration, membrane damage, oxidative stress-mediated apoptosis (enhanced by gastric fluid transformation)	16	Tier 1
	Banerjee et al. (2022)	HepG2	0.1–100 μg/mL	1–24 h	Particle size/functionalization/concentration/time-dependent hepatocyte toxicity	18	Tier 1
	Huang et al. (2024)	HepG2	The concentration of TPHP was diluted with DMSO to 0, 10, 50, 100, and 200 nmol/mL, and the concentration of PS-NPs/MPs was 50 μg/mL individually	24 h	Oxidative stress, ΔΨm dysfunction, LDH release, apoptosis (potentiated by TPHP co-exposure)	17	Tier 1
	Sun H. et al. (2023)	HepG2	22.3, 48.6, 105.6, 230.0, and 500.0 μM of bisphenol A or Pb(NO3)2, or with PS-NPs of 116.2, 250.0, 538.9, 1,160.9, and 2,500.9 μg/mL	30 h	Additive toxicity with bisphenol A and lead	18	Tier 1
	Rosellini et al. (2024)	CYP3A4-over-expressing HepG2 cells	Cells were treated with different compounds ranging from 0.003 to 100 µM in a total volume of 200 µL	72 h	Dysregulated mitotic/DNA-replication and metabolic/inflammatory pathways; hepatotoxicity	18	Tier 1
Lung	Li et al. (2023f)	16HBE cells	0, 1 and 100 μg mL−1 of the photoaging TWPs samples	6 h, 24 h and 72 h	EPFR generation on TWP surface, enhanced by photoaging	18	Tier 1
	Roshanzadeh et al. (2020)	A549 cells	PS-NPs and amine-functionalized NPs: 100 μg/mL	0, 5, 15, 30, and 60 min	Surface charge-dependent uptake/toxicity under dynamic breathing-relevant conditions	17	Tier 2
	Yang et al. (2021)	HPAEpiC and BEAS-2B cells	0, 2.5, 5, 7.5, 10, 15, 20, 25, 30,35 and 40 μg/cm2	24 h	Tight junction disruption, redox imbalance, inflammation, apoptosis	18	Tier 1
	Wu et al. (2024b)	BEAS-2B cells	PS-MPs:0.05, 0.15, 0.2 mg/mL PS-NPs: 0.05, 0.15, 0.2 mg/mL	24 h	Oxidative stress, apoptosis, ferroptosis (GPX4/FTH1 reduction), mitochondrial alteration, elevated MDA	17	Tier 1
	Milillo et al. (2024)	A549 cells	PS-NPs:10–25–50–100–250–500 μg/mL	24 h, 48 h and 96 h	Reduced viability, oxidative stress, senescence, apoptosis, cytoplasmic penetration	18	Tier 1
	K C et al. (2023)	A549 cells	PTFE-MPs: 0,10, 100, 500, 1,000 μg/mL	24 h	NO/ROS production, TNF-α/IL-6 secretion, MAPK activation (no direct cytotoxicity)	17	Tier 1
	Xu et al. (2019)	A549 cells	PS-NP25: 30, 25, 20, 15, 10, 5, and 2.5 μg/mL PS-NP70: 300, 220, 160, 100, 60, 30, and 10 μg/mL	24 h	Reduced viability, cell cycle S-phase arrest, inflammatory gene activation, pro-apoptotic protein changes	18	Tier 1
	Ji et al. (2021)	A549 cells	PS, PS@Bap and PS@Bap@Mucin: 10, 20, 50, 100 and 150 μg/mL	30 min, 3, 6, 12 and 24 h	Protein corona-modulated uptake, trafficking, cytotoxicity	17	Tier 1
	Liu et al. (2024)	BEAS-2B cells	PVC-MPs: 100, 200, 400, 600 and 800 μg/mL	24 h	Reduced viability; altered fluid shear stress/MAPK/TGF-β gene expression and amino acid metabolism	15	Tier 1
	Halimu et al. (2022)	A549 cells	PS-NP20 +: 10, 20 and 40 μg/mL PS-NP20: 10, 20 and 40 μg/mL PS-NP50: 40, 80 and 160 μg/mL	24 h	Increased migration, EMT markers, ROS, NADPH oxidase	15	Tier 1
	Huang et al. (2022)	HNEpCs cells	MNPs: 10, 50, 125, 500 e 1250 μg/mL	48 h	Inhibited cell growth/proliferation	16	Tier 1
	Zhang, H. et al. (2022)	A549 cells	Nano-PET: 0.1–196.79 μg/mL	24 h	Hormetic effect: mild proliferation at low dose, inhibition at high dose	16	Tier 2
Kidney	Wang, Y. L. et al. (2024)	HK-2 cells	0, 04 and 0.8 mg/mL	24 h	Increased EV production, ER stress (without inflammation)	15	Tier 1
	Zhu et al. (2023)	HK-2 cells	0,50, or 100 μg/mL	12 h	MAPK-mediated apoptosis, ROS, mitochondrial/ER structural changes	16	Tier 1
	Li et al. (2023d)	293 T cells	1, 5, 25, 75, and 125 μg/mL	12 h	Size-dependent internalization and cytotoxicity (LO2 more resistant than 293 T)	16	Tier 1
	Li et al. (2023b)	HEK293 cells	4-PBA: 10, 20, 50, 100, 200, 400 μM Then treated with PS-NPs 50 μg/mL or LPS 5 μg/mL or combined treatment of PS-NPs and LPS	12 h 4-PBA 24 h PS-NPs or 12 h LPS or PS-NPs and LPS,PS-NPs for 12 h	IRE1/XBP1 ER-stress pathway and apoptotic factor inhibition (with 4-PBA)	16	Tier 1
	Chen et al. (2022)	HEK293 cells	3–300 ng/mL	24 h	Oxidative stress *via* HO-1 inhibition, mitochondrial depolarization, autophagy, apoptosis	16	Tier 1
	Zhou et al. (2024)	hiPSCs	0, 0.625, 1.25, 2.5, 5, 10, and 20 μg/mL	24, 48, 72, 96, 120, and 240 h	Reduced organoid size/nephron structure, ROS-mediated apoptosis	15	Tier 1
	Wang, Y. L. et al. (2021)	HK-2 cells	0.025, 0.05, 0.1, 0.2, 0.4, or 0.8 μg/mL	5, 10, 30, 60 and 120 min	Mitochondrial ROS, Bad expression, MAPK/AKT/mTOR pathway alteration	15	Tier 1
Reproductive and breast	Wang, W. et al. (2024)	KGN cells	NPs: 0, 50, 100, 150, 200, and 250 μg/mL TCS: 0, 5, 15, 25, 35, and 45 μM	24 h	Increased ROS and MDA (co-exposure)	18	Tier 1
	Qin et al. (2024)	Human endometrial organoids	5 and 50 μg/mL of MPs	48 h	Apoptosis, disrupted growth (endometrial organoids)	18	Tier 1
	Zeng et al. (2023)	KGN cells	100 μg/mL of PS-NPs and 400 μM of salidroside	48 h	Inhibited proliferation, apoptosis, ROS, Hippo pathway activation (MST1/LATS1/YAP1), reduced CTGF/Cyr61	15	Tier 1
	Pontecorvi et al. (2023)	VK2 E6/E7 cells	PE N/MPL: 25 and 250 μg/mL	48, 72 and 120 h	Altered junctional/adherence proteins and actin cortex; oxidative stress and barrier-related gene/miRNA changes	18	Tier 2
	Huang et al. (2023b)	COV434 cells	Nano-PS: 50, 100, 150 and 200 μg/mL	24 h	Reduced viability/MMP, increased apoptotic/oxidative markers, cell cycle arrest	18	Tier 1
	Zhong et al. (2023)	HeLa cells	NPs: 1; 10, 50; 100 mg/L V6: 5; 10; 25; 50 mg/L	4 h	ROS-mediated apoptosis gene regulation	17	Tier 1
	Schirinzi et al. (2017)	HeLa cells	from 10 ng/mL to 10 μg/mL	24–48 h	Oxidative stress-mediated cytotoxicity	15	Tier 1
	Park J. H. et al. (2023)	MCF-7 and MDA-MB-231 cells	PPMPs: 0, 0.4, 0.8, 1.2, 1.6 and 2.0 mg/mL	24 h	Enhanced metastasis-related gene/cytokine expression	17	Tier 1
	Li, N. et al. (2024)	spermatozoa	Microplastics identified in this study were: ABS, PET, PE, PC, PP, PS, PVC and PTEF.	​	Semen contamination (non-occupational exposure)	16	Tier 1
Gastric	Qin et al. (2022)	GES-1 cells	pristine PS-MPs L-Cl_2_-PS-MPs and H-Cl_2_-PS-MPs 1, 10, 20, 50, and 100 mg/L	48 h	PI3K/AKT, Bcl-2/Bax, caspase cascade-mediated apoptosis	16	Tier 1
	Ding et al. (2021)	GES-1 cells	PS-NPs: 50 g/mL; 500 μg/mL	2, 4, 6, 12, 24, and 48 h	Endocytosis-pathway-dependent uptake (caveolae/clathrin/macropinocytosis/RhoA/actin)	18	Tier 1
	Banerjee A et al. (2021)	SNU-1 cells	80 μL of particle stocks (50–5,000 nm aminated, carboxylated, or NF particles) in RPMI containing 0.225, 2.25, 22.5 or 225 μg/mL particles and 0.0056% v/v Tween 20 were added to each well to obtain final particle concentrations of 0.1, 1, 10, 100 μg/mL, respectively and 0.0025% v/v Tween 20	1, 2, 4, 6 or 24 h	Particle size/dose/functionalization/exposure-dependent toxicity	15	Tier 2
	Yan et al. (2020)	AGS cells	PS-NPs, PS-MPs: 50, 100, 200, 400, 600 mg/L TC: 0.5, 1, 5, 10, 20 mg/L	24 h	Concentration-dependent adsorption	16	Tier 1
	Lin et al. (2021a)	AGS cells	8 μg/mL 20 nm PS (PS20), 128 μg/mL 50 nm PS (PS50), 128 μg/mL 200 nm PS (PS200), 128 μg/mL 500 nm PS (PS500), 128 μg/mL 1,000 nm PS (PS1000), 20 μM A (As), 128 μg/mL 20 nm PS + 20 μM A (PS20-As), 128 μg/mL 50 nm PS + 20 μM A (PS50-As), 128 μg/mL 200 nm PS + 20 μM A (PS200-As), 128 μg/mL 500 nm PS +20 μM A (PS500-As) and 128 μg/mL 1,000 nm PS+ 20 μM A (PS1000-As)	24 h	Enhanced arsenic cytotoxicity/accumulation	18	Tier 1
	Han W. et al. (2024)	Human gastric fibroblasts cells (HGF)	0 mg/L, 1.5 mg/L, 15 mg/L, 150 mg/L	24 h	Size- and concentration-dependent uptake, apoptosis/necrosis	15	Tier 1
	Lin et al. (2021b)	AGS cells	PS: 0, 0.5, 1, 2, 4, 8, 16, 32, 64 μgmL−1 or OA: 0, 5, 10, 20, 40, 60, 80 ng mL−1	24 h	Reduced viability, mitochondrial depolarization, reduced IL-10/p53, increased ROS/Ca2+/IL-8	18	Tier 1
Brain	González-Caballero et al. (2024)	hNS1 cells	PS-NPs: 0.5, 2.5, and 10 mg/L	1, 2 or 4 days	Cellular uptake, aggregation, apoptosis, reduced proliferation	16	Tier 2
	Bai et al. (2024)	hCMEC/D3 cells	APS-NPs: 25 and 50 mg/mL Camellia pollen: 2.5–40 μg/mL	12 h	Tight junction disruption *via* TLR2/MMP9, BBB permeation (mitigated by Camellia pollen)	17	Tier 1
	Tang et al. (2022)	SHSY-5Y cells	PS-NPs: 20, 50, 100, 200, and 500 mg/L	24 h	Mitochondrial damage, reduced proliferation, LDH release, oxidative stress, apoptosis	15	Tier 1
	Martin-Folgar et al. (2024)	hNS1 cells	NPs: 0.5, 2.5 and 10 μg/mL	4 days	Oxidative stress, DNA damage, inflammation, apoptosis	18	Tier 1
	Schirinzi et al. (2017)	T98G cells	from 10 ng/mL to 10 μg/mL	24–48 h	Oxidative stress-mediated cytotoxicity	18	Tier 1
Heart	Li, J. et al. (2024)	Human ESC lines H1 and hESC-derived cardiac organoids	PS-NPs: 5 μg/mL, 20 μg/mL	24 or 48 h	Impaired cardiomyocyte differentiation/maturation, reduced organoid contractility	15	Tier 1
	Zhou et al. (2023)	Human pluripotent stem cell-derived *in vitro* three-dimensional cardiac organoid (CO)	0, 0.025, 0.25 and 2.5 μg/mL	48 or 72 h	Oxidative stress, inflammation, apoptosis, collagen accumulation	15	Tier 1
Skin	Han W. et al. (2024)	HaCaT cells	10, 50, and 100 μg/mL	24–72 h	Increased skin cell inflammation	16	Tier 1
	Lihua and Zhiyin (2023)	FLS cells	0–1.0 mg/mL	60 minut or 48 h	Increased fibroblast-like synoviocyte proliferation	16	Tier 1
	Peng et al. (2024)	BJ-5ta cells	PS-NPs: 0.001, 0.01, 0.1, 1 and 10 μg/mL	72 h	Time-, size- and concentration-dependent inhibition of fibroblast migration	16	Tier 1
	Gopinath et al. (2021)	HaCaT cells	PENPs, PSNPs, NPs-1, NPs-2 and H_2_O_2_ : 0, 25, 50, 100, 250 and 500 μg/mL	24, 48, 72, 96, 120 and 144 h	Oxidative stress-mediated reduced proliferation/growth	15	Tier 1
Bone marrow and blood	Najahi et al. (2022)	BMMSCs and AMSCs cells	MPs-PET<1 and <2.67 μm	24, 48 and 72 h	Altered stem cell differentiation potential	15	Tier 1
	Ilić et al. (2022)	Jurkat cells	AgNP: 0.1, 1, 10, 25, 50 and 100 mg/mL AgNO_3_ : 1.7, 4.2, 8.5, 12.7, and 17.0 mg/mL PSNP: 1, 10, 50, 100, 250 and 500 mg/mL	24 h	Additive oxidative stress, apoptosis, reduced cell stiffness (AgNP + PSNP)	16	Tier 2
	K C et al. (2023)	THP-1 and Jurkat cells	PTFE-MPs: from 1 to 1,000 μg/mL	48 h	NO/ROS production	16	Tier 1
	Chen et al. (2023)	EA.hy926 human vascular endothelial cells	PSMPs: 0.04 μg/mL, 4.00 μg/mL, and 40.00 μg/mL	24 h	Reduced ROCK1/NF-κB/NLRP3 expression	16	Tier 1
	Kik et al. (2021)	PBMCs cells	PS-NPs: 0.001–100 μg/mL	24 h	Redox imbalance, lipid/protein oxidation	18	Tier 1
Blastocyst and placenta	Wei et al. (2022)	HUVECs cells	PS nanoplastic 0.05, 0.1, 0.25 and 0.5 mg/mL	1, 3 or 6 h	Disrupted VE-cadherin junctions	15	Tier 1
	Lee et al. (2021)	HUVECs cells	PS-MPs: 0, 20, 40, 60, 80, and 100 μg/mL	24, 48, and 72 h	Reduced viability; dose/size-dependent accumulation	17	Tier 2
	Wan et al. (2024)	Human trophoblast Swan 71 cells	50, 100, 150, or 200 μg/mL PS-NPs	24 h	Oxidative stress, reduced MMP, Bcl-2/caspase-mediated apoptosis	15	Tier 1
	Shen et al. (2022)	human placental choriocarcinoma cells (JEG-3)	PS-NPs: 0, 20, 39, 78, 156, 313, 625, 1,250, 2,500 and 5,000 μg/mL	24 h	Size-dependent placental cell toxicity	17	Tier 1
	Zhang M. et al. (2022)	Human umbilical vein endothelial cells (HUVECs)	MPs 1,000 μgmL−1	24 h	Reduced viability/migration/tubule formation, apoptosis, ROS (size-dependent)	16	Tier 1

### Gut

Several studies used Caco-2 cells, a human colon epithelial adenocarcinoma cell line, to evaluate the impact of various microplastic types and exposure conditions (Ding et al., 2022; Domenech et al., 2021; Huang W. et al., 2021; Yan L et al., 2023; Zhang Y. et al., 2022). For instance, Jin et al. (2024) exposed Caco-2 cells to a 9:1 mixture of medium and raw leachate/supernatant/resuspended MPs, observing significant oxidative stress, altered intracellular redox states, and aggravated metabolic dysfunction after 48 h. Similarly, Stock et al. (2022) evaluated the impact of different concentrations (ranging from 5 × 10^9^ to 1 × 10^12^ particles/mL) on Caco-2 and HT29-MTX-E12 cells, finding that properties like particle size and surface chemistry influenced the cellular uptake and toxicity of micro- and nanoplastics (NPs).

Beyond their combined effects with other pollutants, nanoparticles alone have also been shown to elicit marked toxicological effects *in vivo*, including hematological and hepatic alterations (Akhtar et al., 2025), which provides a useful frame of reference for interpreting their interaction with MPs described below.

A number of studies also explored the combined effects of MPs and other pollutants. For example, Ding et al. (2024) investigated the interaction of PS-MPs with nanoparticles (NPs) and detected oxidative stress, inflammatory lesions, and activation of the MAPK signaling pathway, leading to apoptosis in Caco-2 cells after both short- and long-term exposures (6 h–6 days). Moreover, the work by Li et al. (2023d) demonstrated that the absorption of PS-NPs in Caco-2 cells further was increased when combined with food matrices, leading to higher levels of cellular inflammation, stress, and apoptosis after 72 h of exposure.

Organoid models were also utilized to assess the impact of MPs, such as those used by Park S. B. et al. (2023) and Hou et al. (2022), both found that microplastic exposure decreased viability, induced gene expression related to inflammation, and caused apoptotic effects in intestinal organoids. Wang et al. (2020) found an alteration of the mitochondrial membrane potential due to PS exposure. Lastly, Liu et al. (2022) documented ROS and MMP changes with PS and PFOS treatments, while Busch et al. (2021) highlighted PE particle-induced cytotoxicity and inflammatory responses in Caco-2 and HT29 cells.

Taken together, these gastrointestinal studies converge on oxidative stress, NF-κB/MAPK-mediated inflammatory signalling, and mitochondrial-dependent apoptosis as recurrent mechanistic themes, although effect magnitude varied considerably with polymer type, particle size, and co-exposure conditions; findings should therefore be interpreted as mechanistically convergent rather than directly comparable across studies.

### Liver

Huang et al. (2023a) reported that exposure to polystyrene (PS) particles of 20 nm size resulted in a dose-dependent decrease in cell viability in HepaRG cells, with higher concentrations leading to significant toxicity. Similarly, Li et al. (2023a) observed that exposure of human normal liver cells (LO2 cells) to NPs of varying sizes (20–1,000 nm) resulted in size- and concentration-dependent toxicity, and the smaller particles increase cell mortality. Stock et al. (2022) highlighted that factors like particle size, surface modification, and hydrophobicity influence cellular uptake and toxicity in HepaRG cells. Further studies, such as those by He et al. (2024), show that PS MPs of various sizes (20–500 nm) induce mitochondrial damage, with 50 nm particles triggering apoptosis in HepG2 cells. Other studies indicate that exposure to MPs leads to elevated calcium levels, oxidative stress, and activation of apoptotic pathways, with Li et al. (2021c) demonstrating that microplastic exposure in L02 cells significantly increased calcium concentrations and activated store-operated calcium channels (SOCs). Additionally, Li Y et al. (2023e) identified that exposure to polystyrene nanoparticles caused mitochondrial injuries, including decreased ATP production and loss of mitochondrial membrane potential in HepG2 cells. Overall, the studies provide compelling evidence that MPs, particularly PS particles, can induce various toxic effects in liver cells, including mitochondrial dysfunction, oxidative stress, and apoptosis. The extent of toxicity is influenced by factors such as particle size, concentration, and exposure duration (Li et al., 2021a; Li et al., 2021b; Ma et al., 2024; Pan et al., 2021). These findings underscore the need for further investigation into the long-term health implications of microplastic exposure in liver function.

Li et al. (2021b) also studied L02 cells at a concentration of 0.5 mg/mL of micro-PS and 100 μM of NaHS for 24 h, finding that H2S suppressed inflammation, apoptosis, and oxidative stress induced by PS MPs and facilitated the translocation from the cytoplasm and nuclear accumulation of Nrf2, leading to a positive regulation of the expression of HO-1 and NQO1 expression. Zheng et al. (2023) combined PS exposure with typical pollutants (BPA, PCP, and Pb(NO3)2) at concentrations ranging from 22.3 to 1,160.9 μg/mL for 48 h and found that PS exhibited additive effects when combined with these types of pollutants. Wang L. et al. (2021) exposed SMMC-7721 cells to PS-MPs at concentrations ranging from 2 to 100 μg/mL for 24 h, noting that the transformation of PS MPs by simulated gastric fluid exacerbated their adverse effects, including morphological changes, membrane damage, and increased apoptosis due to oxidative stress. Banerjee et al. (2022) studied HepG2 cells with PS particles at concentrations between 0.1 and 100 μg/mL for 1–24 h, confirming that PS particles could be detrimental to hepatocytes, with the extent of toxicity depending on particle size, surface functionalization, concentration, and exposure time. Huang et al. (2024) investigated the co-exposure of HepG2 cells to PS-NPs/MPs (50 μg/mL) and TPHP (0, 10, 50, 100, and 200 nmol/mL) for 24 h, revealing that the co-exposure of PS-NPs/MPs induced more potent oxidative stress, mitochondrial dysfunction, lactate dehydrogenase (LDH) release, and apoptosis compared with single TPHP exposure. Sun H. et al. (2023) studied the effects of PS-NPs in combination with bisphenol A and lead at concentrations of 116.2–2,500.9 μg/mL for 30 h and found additive effects on cellular toxicity. Lastly, Rosellini et al. (2024) exposed CYP3A4-over-expressing HepG2 cells to various compounds ranging from 0.003 to 100 µM for 72 h and identified pathways related to mitosis and DNA replication that were suppressed, alongside other mis-regulated metabolic and inflammation-related pathways, suggesting the induction of hepatotoxicity.

In addition to polystyrene, other polymer types have also been implicated in hepatotoxicity: evaluation of polyvinyl chloride microplastics in ducks revealed oxidative and fibrotic hepatic outcomes (Chen et al., 2025), supporting the relevance of PVC alongside PS in liver-related MNP toxicity. Beyond plastic particles themselves, co-occurring environmental pollutants such as nickel have also been shown to induce hepato-intestinal injury and dysregulation of conjugated bile acids in mice (Yuan et al., 2025), highlighting shared target organs between MNPs and other emerging contaminants.

### Lung

Li et al. (2023b) observed that the photoaging of tire wear particles (TWPs) in samples exposed to 16HBE cells enhances the formation of environmentally persistent free radicals (EPFRs), suggesting increased toxicity potential over time. Studies on other lung cell types, such as A549 cells, reveal that the uptake and toxicity of polystyrene nanoplastics (PS-NPs) are influenced by particle surface charge, an important factor under dynamic conditions simulating breathing exposures (Milillo C et al., 2024; Roshanzadeh et al., 2020). Yang et al. (2021) found that exposure to PS-NPs in HPAEpiC and BEAS-2B cells reduces transepithelial electrical resistance and disrupts tight junction proteins, triggering a redox imbalance and apoptotic. Further investigations, such as those investigated by Wu et al. (2024), demonstrated that exposure to PS-NPs in BEAS-2B cells induces oxidative stress and ferroptosis through the reduction of key proteins such as GPX4 and FTH1. Additionally, PTFE-MPs (polytetrafluoroethylene microplastics) have been associated with the activation of MAPK signaling pathways and the production of nitric oxide and reactive oxygen species, without direct cytotoxic effects (K C et al., 2023). Xu et al. (2019) found that PS-NPs significantly affect A549 cell viability, causing S-phase cell cycle arrest, activate inflammatory genes, and alter apoptosis-associated proteins. Ji et al. (2021) found that a protein corona modified with PS, PS@Bap, and PS@Bap@Mucin impacts cellular uptake and intracellular trafficking, influencing cytotoxicity in A549 cells. In a study on BEAS-2B cells, Liu et al. (2024) observed that PVC-MPs decrease cell viability and modulate gene expression related to MAPK and TGF-β signaling pathways, as well as amino acid metabolism.

Further findings include the impact of PS-NPs on cellular functions, with Halimu et al. (Halimu et al., 2022) showing that PS-NPs promote migration in A549 cells and elevate EMT markers alongside increased reactive oxygen species and NADPH oxidase levels. Huang et al. (Huang et al., 2022) investigated the effects caused by different sizes and PS particleson HNEpC cells, noting that most micro- and NPs significantly inhibited cell proliferation within 24 h, with stable viability curves after 48 h. Finally, Zhang H. et al. (2022) reported a biphasic response of Nano-PET exposure in A549 cells, where low concentrations slightly promoted cell viability, while higher concentrations caused an inhibitory effect, highlighting concentration-dependent toxicity.

Overall, pulmonary *in vitro* evidence converges on oxidative stress, surface-charge-dependent internalization, and MAPK/ferroptosis-related apoptotic signalling as recurrent themes, although reliance on submerged monoculture systems rather than air-liquid interface models limits extrapolation to realistic inhalation exposure.

### Kidney

Wang Y-L. et al. (2024) reported that exposure to polystyrene microplastics (PS-MPs) in HK-2 cells enhanced extracellular vesicle production and activated proteins related to endoplasmic reticulum (ER) stress, though inflammation-related proteins were not induced, suggesting a selective response to MPs. Zhu et al. (2023) found that PS-NPs exposure in HK-2 cells triggered apoptosis through the MAPK signaling pathway, promoted reactive oxygen species (ROS) production, and caused structural alterations in the mitochondria and ER. This evidence supports the role of MPs in disrupting mitochondrial and ER integrity, which are essential for renal cell function.

Li et al. (2023d) further examined nanoparticle size effects, noting that smaller PS-NPs elevated cell mortality rates in 293T cells. Interestingly, despite greater internalization in LO2 cells, these cells demonstrated higher resistance to PS-NPs than 293T cells, suggesting that different cell types exhibit varying resilience to microplastic-induced toxicity. Li et al. (2023e) also showed that pre-treatment with 4-phenylbutyric acid (4-PBA) inhibited the ER stress-related apoptosis process in HEK293 cells co-exposed to PS-NPs and lipopolysaccharide (LPS), implicating ER stress pathways in MP toxicity.

Chen et al. (Chen et al., 2022) and Zhou et al. (Zhou et al., 2024) identified the key role of oxidative stress and apoptosis after exposure to PS-MPs in HEK293 and hiPSCs cells, respectively. PSMPs inhibited antioxidant pathways in HEK293 cells, leading to mitochondrial membrane depolarization and autophagosome formation, signifying that both autophagy and apoptosis may be concurrently triggered by MPs. In kidney organoids derived from hiPSCs, Zhou et al. (Zhou et al., 2024) observed that both short- and long-term exposure reduced organoid size and disrupted nephron structure, alongside increased ROS levels and apoptosis, indicating a particular vulnerability of early kidney development to MP toxicity.

Supporting these findings, *in vivo* evidence further confirms renal toxicity of microplastics. Ijaz et al. (Ijaz et al., 2024) reported that polystyrene microplastic exposure induced significant renal toxicity in rats, while treatment with robinetin showed ameliorative effects against oxidative stress-mediated kidney damage, highlighting both the toxic potential of MPs and possible protective interventions.

Finally, Wang Y-L. et al. (2021) showed that PS-MPs uptake in HK-2 cells resulted in elevated mitochondrial ROS and the mitochondrial protein Bad. They also observed modifications in the phosphorylation of MAPK and AKT/mTOR signaling pathways, underlining the involvement of these key regulatory pathways in the cellular response to MPs. Collectively, these studies underline that MPs elicit diverse cytotoxic responses in kidney cells, mediated by oxidative stress, apoptosis, and ER stress pathways, which could have implications for kidney health, especially under chronic exposure scenarios. However, direct comparison across studies remains limited by heterogeneous exposure metrics (mass, particle number, or surface-area concentrations) and inconsistent particle characterization.

### Reproductive and breast

Wang W. et al. (2024) found that co-exposure to nanoparticles (NPs) and triclosan (TCS) in KGN cells elevated reactive oxygen species (ROS) and malondialdehyde (MDA), lipid oxidation product, indicating oxidative damage. Qin et al. (Qin et al., 2024) reported apoptotic responses and disrupted growth patterns in human endometrial organoids exposed to MPs, suggesting significant impacts on endometrial cell health and development.

Zeng et al. (Zeng et al., 2023) observed that PS-NPs exposure in KGN cells inhibited cell proliferation, induced apoptosis, accumulated ROS, and activated Hippo pathway regulators (MST1, LATS1, and YAP1), while downregulating mRNA levels of CTGF and Cyr61, markers associated with cellular proliferation and survival. This points to a pathway-dependent apoptotic effect of MPs in ovarian cells. In VK2 E6/E7 vaginal epithelial cells, Pontecorvi et al. (Pontecorvi et al., 2023) showed that internalized N/MPLs altered junctional proteins, actin organization, and oxidative stress-related gene expression, which could compromise epithelial barrier integrity.

Huang et al. (Huang et al., 2023b) documented that Nano-PS exposure in COV434 ovarian cells led to decreased cell viability, reduced mitochondrial membrane potential, elevated apoptotic markers, and induced cell cycle arrest, suggesting the role of oxidative stress in cell cycle disruption. In HeLa cells, Zhong et al. (Zhong et al., 2023) highlighted increased ROS production and apoptosis-related gene expression in response to NPs, while Schirinzi et al. (Schirinzi et al., 2017) confirmed oxidative stress as a primary cytotoxic mechanism in these cells.

Park J. H. et al. (2023) demonstrated that PPMP exposure in breast cancer cells (MCF-7 and MDA-MB-231) upregulated metastasis-related genes and cytokines, potentially exacerbating metastatic activity in breast cancer.

Finally in a study by Li N. et al. (2024), researchers identified microplastic contamination in semen samples, even among individuals without occupational exposure. This contamination included various types of MPs, such as acrylonitrile butadiene styrene (ABS), polyethylene terephthalate (PET), polyethylene (PE), polycarbonate (PC), polypropylene (PP), polystyrene (PS), polyvinyl chloride (PVC), and polytetrafluoroethylene (PTEF).

In addition, *in vivo* studies have demonstrated systemic toxic effects of nanomaterials on biological systems. Abduallah et al. (Abduallah et al., 2025) reported that exposure to vanadium, magnesium, and nickel nanocomposites induced significant toxic effects in erythrocytes and reproductive organs of male albino rats, highlighting potential risks of nanomaterial exposure on reproductive health.

Collectively, evidence from ovarian, endometrial, breast, and male reproductive models converges on oxidative stress and mitochondrial-apoptotic signalling as shared mechanisms, but the heterogeneity of cell models and exposure paradigms limits direct cross-study comparison and calls for standardized reproductive toxicity endpoints in future work.

### Gastric

Several studies have explored the effects of MPs and NPs on gastric cells, highlighting various cellular responses based on particle type, size, concentration, and exposure time. Qin, J. et al. (Qin J et al., 2022) found that chlorinated polystyrene microplastics (PS-MPs) in GES-1 cells triggered apoptosis through oxidative stress, mitochondrial depolarization, and the activation of the tumoral (PI3K/AKT) and apoptotic (Bcl-2/Bax) pathways. Similarly, Ding, Y. et al. (Ding et al., 2021) observed that PS nanoparticles (PS-NPs) enter GES-1 cells *via* multiple endocytotic pathways, with certain inhibitors reducing this uptake. Banerjee, A. et al. (Banerjee A et al., 2021) demonstrated that the cytotoxicity of micro- and nanoparticles in SNU-1 gastric cells varies with particle size, concentration, surface functionalization, and exposure duration, revealing dose-dependent toxicity. Yan, X. et al. (Yan et al., 2020) showed that PS particles increase adsorption with rising concentrations of TC, which may lead to increased toxic effects. Lin, P. et al. (Lin et al., 2021a) found that subcytotoxic concentrations of PS enhanced the accumulation and toxicity of arsenic in AGS cells, exacerbating cytotoxicity. Moreover, Han M. et al. (2024) reported that smaller PS particles were more readily incorporated by human gastric fibroblast cells (HGF), resulting in cell death through both apoptotic and necrotic pathways, dependent on particle size and concentration. Lastly, Lin, P. et al. (Lin et al., 2021b) identified combined toxicity effects of PS and oleic acid (OA) on AGS cells, evidenced by decreased cell viability, mitochondrial membrane depolarization, and increased oxidative stress, intracellular ROS, calcium levels, and pro-inflammatory markers like IL8.

### Brain

Research on the impact of MPs and NPs on brain cells reveals significant toxicity mechanisms and cellular responses. González-Caballero, M. C. et al. (González-Caballero MC et al., 2024) reported that polystyrene nanoparticles (PS-NPs) entered human neural stem cells (hNS1), likely through endocytosis, and accumulated in the cytoplasm, leading to apoptosis and reduced cell proliferation, though not penetrating the nucleus. Bai, H. et al. (Bai et al., 2024) found that aminated PS-NPs (APS-NPs) disrupted tight junction proteins (Occludin and ZO-1) in hCMEC/D3 cells through the TLR2/MMP9 axis, compromising the blood-brain barrier (BBB). Treatment with Camellia pollen partially alleviated these effects, suggesting a potential protective intervention. Tang, Q. et al. (Tang et al., 2022) showed that PS-NPs exposure in SHSY-5Y cells resulted in mitochondrial damage, reduced proliferation, increased oxidative stress, calcium influx, apoptosis, and decreased mitochondrial membrane potential and ATP levels. Martin-Folgar, R. et al. (Martin-Folgar R et al., 2024) demonstrated that exposure to nanoparticles in hNS1 cells led to oxidative stress, DNA damage, altered inflammatory response, and apoptosis, suggesting potential links to neurodevelopmental diseases. Finally, Schirinzi, G. F. et al. (Schirinzi et al., 2017) confirmed that oxidative stress plays a key role in nanoparticle-induced cytotoxicity in T98G brain cells. These studies collectively highlight the potential ability of MPs and NPs to impair cellular function and integrity in brain cells, indicating risks for neurotoxicity and neurological health.

### Heart and skin

For heart cells, Li J. et al. (2024) observed that exposure to polystyrene nanoparticles (PS-NPs) in human embryonic stem cells (ESCs) and cardiac organoids led to compromised cardiomyocyte differentiation, resulting in immature cardiomyocytes and smaller cardiac organoids with impaired contractility. Zhou, Y. et al. (Zhou et al., 2023) demonstrated that human pluripotent stem cell-derived three-dimensional cardiac organoids exposed to low concentrations of PS-NPs (0–2.5 μg/mL) for up to 72 h exhibited increased oxidative stress, inflammation, apoptosis, and collagen accumulation, indicating adverse effects on cardiac tissue formation and function.

In skin cells, Han M. et al. (2024) reported that nano-sized MPs increased inflammation levels in HaCaT cells (human keratinocytes) after 24–72 h of exposure to concentrations ranging from 10 to 100 μg/mL. Lihua, C. et al. (Lihua and Zhiyin, 2023) found that MPs promoted the proliferation of rheumatoid arthritis fibroblast-like synoviocytes (RA-FLSs) in a dose- and time-dependent manner. Peng, M. et al. (Peng et al., 2024) showed that PS-NPs (ranging from 0.001 to 10 μg/mL) inhibited fibroblast migration, with larger particles at higher concentrations causing more significant effects. Finally, Gopinath, P. M. et al. (Gopinath et al., 2021) revealed that both single-dose and chronic exposure to various types of nanoparticles, including PS-NPs, resulted in oxidative stress-mediated inhibition of cell growth and proliferation in HaCaT cells, demonstrating the long-term detrimental effects on skin cell function. These studies highlight the cellular dysfunction induced by microplastic and nanoparticle exposure in both heart and skin cells, contributing to oxidative stress, inflammation, and impaired cell function.

### Bone marrow and blood

Research on the impact of MPs and NPs on bone marrow and blood cells has shown that exposure to these particles induces cellular stress, apoptosis, and alterations in cell function.

In bone marrow mesenchymal stem cells (BMMSCs) and adipose mesenchymal stem cells (AMSCs), Najahi, H. et al. (Najahi et al., 2022) found that exposure to PET MPs with particle sizes under 2.67 μm for up to 72 h altered their differentiation potential. This suggests that microplastic exposure could interfere with the normal regenerative functions of stem cells in the bone marrow.

Ilić, K. et al. (Ilić et al., 2022) observed that exposure to silver nanoparticles (AgNPs), polystyrene nanoparticles (PSNPs), and silver nitrate (AgNO3) induced oxidative stress, apoptosis, and reduced cell stiffness in Jurkat cells (human lymphocytes) after 24 h. This highlighted the toxic effects of these nanoparticles on immune cells, particularly in terms of cellular structural integrity and stress responses. Similarly, K C et al. (2023) showed that polytetrafluoroethylene microplastics (PTFE-MPs) induced the production of nitric oxide and reactive oxygen species (ROS) in THP-1 and Jurkat cells after 48 h, indicating a potential inflammatory and oxidative damage in blood cells. Chen, Y. C. et al. (Chen et al., 2023) found that polystyrene microplastics (PSMPs) triggered apoptosis in human vascular endothelial cells (EA.hy926) and reduced the expression of signaling molecules such as Rho-associated protein kinase-1, contributing to disrupted cellular functions. Kik, K. et al. (Kik et al., 2021) further demonstrated that PS-NPs disrupted the redox balance in peripheral blood mononuclear cells (PBMCs), leading to elevated ROS levels and increased lipid and protein oxidation after just 24 h of exposure, highlighting the oxidative damage induced by these particles.

These findings underscore the harmful effects of MPs and NPs on both bone marrow and blood cells, with potential implications also in immune response, cell regeneration, and overall tissue health.

### Blastocyst and placenta

Wei et al. (Wei et al., 2022) exposed HUVECs (human umbilical vein endothelial cells) to PS NPs at concentrations of 0.05, 0.1, 0.25, and 0.5 mg/mL for 1, 3, or 6 h. The results revealed that the treatment with PS NPs caused a disruption in vascular endothelial cadherin junctions in a dose-dependent manner, indicating potential alterations in endothelial cell integrity. Similarly, Lee (Lee et al., 2021) investigated the effects of PS-MPs (microplastics) on HUVECs at concentrations ranging from 0 to 100 μg/mL for 24, 48, and 72 h. The study found a significant reduction in cell viability, with intracellular accumulation of PS-MPs observed in a dose- and size-dependent manner. Wan (Wan S et al., 2024) exposed human trophoblast Swan 71 cells to PS-NPs at concentrations of 50, 100, 150, and 200 μg/mL for 24 h. The findings indicated that PS-NPs exposure increased oxidative stress, decreased mitochondrial membrane potential, and induced apoptosis by activating Bcl-2/Cleaved-caspase-2/Cleaved-caspase-3 signaling through the involvement of the mitochondrial pathway. Shen et al. (Shen et al., 2022) exposed human placental choriocarcinoma cells (JEG-3) to various concentrations of PS-NPs (ranging from 0 to 5,000 μg/mL) for 24 h. The results demonstrated that smaller PS-NPs induced greater toxicity in human placental cells, as reported by a decrease in cell viability and an increase in oxidative stress. Finally, Zhang M. et al. (2022) evaluated the effects of MPs (microplastics) on human umbilical vein endothelial cells (HUVECs) at a concentration of 1,000 μg/mL for 24 h. This study found that smaller MPs caused more severe endothelial cell injury, including reduced cell viability, migration, tubule formation, and increased apoptosis and ROS generation.

## Discussion

### Organ-specific synthesis

This systematic review synthesized *in vitro* evidence on apoptosis-related toxicity induced by MNPs across multiple organ-specific cellular models and organoids. Overall, the findings indicate that MNPs can trigger a broad spectrum of cytotoxic responses, with apoptosis emerging as a recurrent endpoint across gastrointestinal, hepatic, pulmonary, renal, reproductive, placental, neural, cardiovascular, skin, and immune-related systems.

Gastrointestinal models were among the most frequently investigated, reflecting ingestion as a major route of exposure. In intestinal cell lines and organoids, MNP exposure was repeatedly associated with oxidative stress, inflammatory responses, altered barrier integrity, mitochondrial dysfunction, and apoptosis-related gene expression. Hepatic models highlighted the liver as a potential target of metabolic and redox-mediated toxicity, with recurrent evidence of mitochondrial damage, altered ATP production, calcium imbalance, and stress-response activation.

Pulmonary models are particularly relevant because inhalation is a major exposure route for airborne microplastics, nanoplastics, and tire-wear particles. Lung epithelial models suggested impairment of barrier properties, modulation of tight-junction proteins, ROS generation, inflammatory signaling, MAPK-related activation, and apoptosis or ferroptosis-associated changes. Renal models and kidney organoids further supported the relevance of mitochondrial and endoplasmic reticulum stress, extracellular vesicle production, oxidative stress, and apoptosis-related pathways.

Reproductive, placental, neural, cardiovascular, skin, and immune-related models broadened the organ-specific picture. Across these systems, MNP exposure was associated with impaired proliferation or differentiation, barrier disruption, oxidative damage, inflammatory activation, mitochondrial dysfunction, and apoptosis-related outcomes. These findings support the interpretation of apoptosis as a shared mechanistic hub rather than an isolated endpoint in a single organ system.

### Convergent molecular pathways and toxicity modifiers

Across organ-specific systems, oxidative stress appears to be the most consistent upstream event. ROS generation may initiate lipid, protein, and DNA damage and activate inflammatory cascades and stress-response signaling. Mitochondrial dysfunction also emerged as a central node, with decreased mitochondrial membrane potential, impaired ATP production, altered mitochondrial morphology, Bcl-2 family modulation, and caspase activation supporting the involvement of the intrinsic apoptotic pathway.

Endoplasmic reticulum stress, calcium imbalance, MAPK activation, PI3K/AKT modulation, Nrf2/HO-1 responses, autophagy, and ferroptosis were also recurrently reported. Rather than acting as independent pathways, these mechanisms likely interact in a dynamic network of stress responses that can converge on apoptosis. Particle size, polymer type, surface charge, surface functionalization, hydrophobicity, aging, protein-corona formation, concentration, exposure duration, and co-exposure with pollutants were important modifiers of cellular uptake and toxicity.

Although this review consistently uses the umbrella term MNPs, the balance of the included *in vitro* evidence remains skewed toward microplastics, with comparatively fewer nanoplastic-specific investigations. Where available, NP-specific findings (e.g., Zeng et al., 2023; Wei et al., 2022; Halimu et al., 2022) were integrated into the organ-specific sections above; however, the smaller nanoplastic evidence base restricts firm conclusions on organ-specific NP toxicity, and this imbalance should be interpreted as a reflection of the current primary literature rather than of the present review methodology.

### Translational relevance and methodological gaps

Although the available evidence supports biological plausibility for MNP-induced apoptosis, translation to real-world human exposure remains limited. Many studies used polystyrene particles, spherical and commercially available particles, short exposure windows, and concentrations that may not fully reflect environmental or occupational exposure scenarios. Future studies should prioritize standardized particle characterization, harmonized concentration metrics, chronic low-dose designs, environmentally realistic mixtures, and advanced human-based models such as organoids, co-cultures, air-liquid interface systems, and microphysiological platforms.

MPs have become significant environmental pollutants, raising concerns about their potential effects on human health. The result of this systematic review has shown that these particles can accumulate in various organs, including the gut, liver, lungs, kidneys, reproductive organs, and gastric cells, leading to a range of cellular dysfunctions in different types of tissues.

In intestinal cells, MP and NP have been shown to cause inflammation and oxidative stress, potentially leading to gastrointestinal disorders. This is consistent with the nanoparticle-specific toxicity reported elsewhere (Akhtar et al., 2025).

Other studies on MPs using Caco-2 and HT-29 cell lines highlight the importance of particle size and surface chemistry in cellular uptake and toxicity. Smaller particles, especially those in the nanometer range, show higher uptake rates and potential for toxicity (Banerjee A et al., 2021; Stock et al., 2022; Wang et al., 2020). Surface functionalization, particularly amination, can enhance uptake and cytotoxicity (Banerjee A et al., 2021). While most studies report low acute toxicity at environmentally relevant concentrations (Stock et al., 2021; Wu et al., 2019), prolonged exposure may lead to mitochondrial dysfunction, and DNA damage (Saenen et al., 2023; Visalli et al., 2021)). MPs can also inhibit efflux pump activity, potentially increasing the toxicity of other substances (Wu et al., 2019). *In vivo* studies suggest limited acute health risks from oral exposure to polystyrene MPs in mammals (Stock et al., 2019).

**FIGURE 3:**
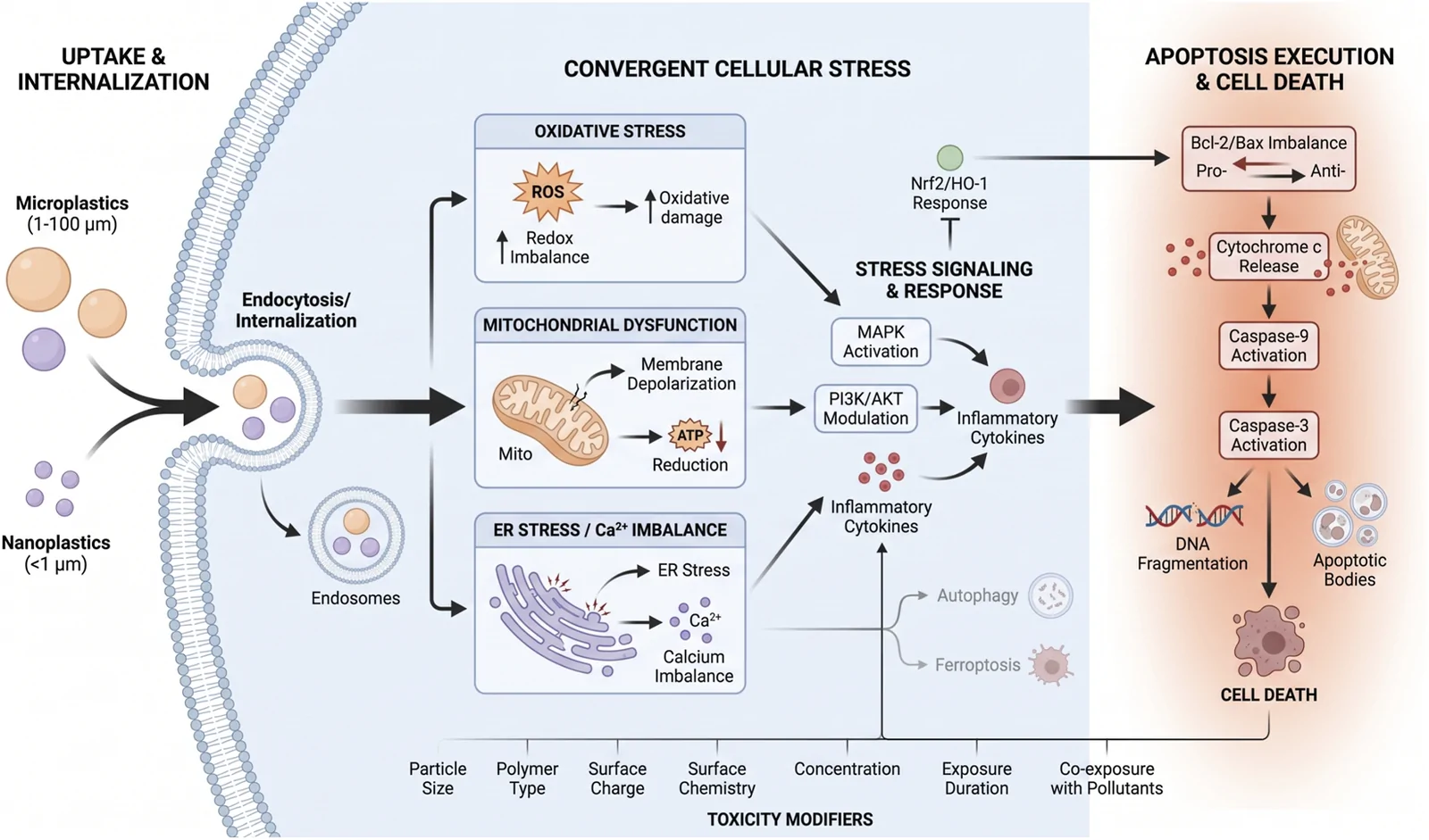
Apoptosis-related molecular pathways activated by micro- and nanoplastics. Micro- and nanoplastics may induce apoptosis through several convergent intracellular mechanisms. These include oxidative stress, mitochondrial dysfunction, calcium imbalance, endoplasmic reticulum stress, MAPK and PI3K/AKT signaling, Nrf2/HO-1 modulation, Bcl-2/Bax dysregulation, cytochrome c release, and caspase activation. These pathways may interact with inflammatory, autophagic, and ferroptotic responses and are influenced by particle characteristics, exposure conditions, and co-exposure with other contaminants. Created in BioRender. Ledda, C. (2026) https://BioRender.com/eprlhyl.

Recent studies have highlighted the detrimental effects of MPs on the digestive system, particularly through oxidative stress, inflammation, and apoptosis (Ding et al., 2024; Piano F 2021). NPs generally induce stronger oxidative stress responses than MPs, while MPs cause more significant alterations in gut microbiota (Kang et al., 2021). The combined effects of MPs/NPs with other pollutants, such as heavy metals and persistent organic pollutants, can exacerbate toxicity through the “Trojan horse” effect (Ledda et al., 2019; Sun R. et al., 2024). This synergistic action can lead to increased inflammation, alteration of the cellular redox state, and apoptosis in intestinal cells (Yan L et al., 2023). The gut microbiota plays a crucial role in mediating the toxicity of MPs/NPs, potentially through the TLR2-My88-NF-κB pathway (Lin et al., 2024) (Figure 3).

MPs modify gut microbiota composition (Li et al., 2020; Qiao et al., 2019) and alter metabolic profiles (Qiao et al., 2019). They can accumulate in the intestine, causing hyperpermeable intestinal barriers and altering gene expression related to intestinal development (Yu et al., 2020). MPs may act as carriers for contaminants like phthalate esters, exacerbating their adverse effects (Deng et al., 2020). These effects have been observed in various models, including mice (Sun N. et al., 2023), zebrafish (Qiao et al., 2019), and nematodes (Yu et al., 2020). The toxicity of MPs in the digestive system is primarily mediated through ROS-induced oxidative stress, leading to various detrimental outcomes (Ding et al., 2023).

MPs in the gut can induce oxidative stress, inflammation, and intestinal barrier dysfunction through various mechanisms. The effects of MP are generated by reactive oxygen species (ROS), leading to mitochondrial damage and depolarization (Das, 2023).

This oxidative stress triggers inflammatory responses and activates pathways like NF-κB/NLRP3/IL-1β/MLCK (Zeng et al., 2024).

The particles PP-NP can disrupt gastric barrier function, inhibit gastric juice secretion, and alter cellular uptake mechanisms (Khan and Jia, 2023; Sun J. et al., 2023). The adverse effects are mediated through multiple pathways, including PI3K/AKT, Bcl-2/Bax, and MAPK signaling cascades (Das, 2023; Jeong et al., 2016).

MPs/NPs exposure can lead to the overproduction of reactive oxygen species, triggering an increase in free radicals involved in inflammatory processes and responsible for disorders in gastric cells (Ding et al., 2023; Hu and Palić, 2020). These particles can induce various forms of programmed cell death, including apoptosis, autophagy, and ferroptosis (Wu et al., 2024a). The harmful effects of MPs/NPs are influenced by factors such as particle size, surface properties, and aging (Kadac-Czapska et al., 2024; Wu et al., 2024a). The modification of the cellular oxidative balance also plays a crucial role in gastric cancer development, affecting initiation, promotion, and progression of cancer cells (Liu et al., 2023).

Natural products have shown potential in restoring oxidative balance and inhibiting Correa’s cascade, a pathological process leading to the development of gastric cancer. Furthermore, *Helicobacter pylori* infection has been linked to chronic gastric diseases mediated to oxidative stress.

Furthermore, other studies have shown that MP toxicity in human cells depends on particle size, concentration, surface functionalization, and exposure duration (Banerjee A et al., 2021; Han M. et al., 2024). Smaller particles are generally more easily uptaken and can induce higher cytotoxicity, and DNA damage (Ding et al., 2023; Wu et al., 2019). However, some studies found larger particles to be more toxic (Visalli et al., 2021). MPs can disrupt mitochondrial function, inhibit efflux pumps, and activate potentially carcinogenic pathways (Ding et al., 2023; Wu et al., 2019). Different cell types show varying sensitivities to MPs, with Caco-2 cells being particularly susceptible (Danopoulos et al., 2022). Adverse effects on cell viability and immune responses have been observed at concentrations as low as 10–20 μg/mL (Danopoulos et al., 2022), highlighting the need for further research on MP toxicity in humans.

Recent studies have explored the effects of MPs on human and animal gut health using advanced models. Human airway and intestinal organoids have demonstrated decreased gene expression, altered cell growth, and inflammatory responses upon MP/NP exposure (Chen et al., 2023; Winkler et al., 2022). In mice, oral exposure to polyethylene MPs altered gut morphology, immune response, and microbiota composition (Djouina et al., 2022). Polystyrene particles induced apoptosis in colon cancer cells through reactive oxygen species production (Wang et al., 2022). In honey bees, MPs exposure decreased gut microbiota diversity and altered gene expression (Wang Y-L. et al., 2021). Human *in vitro* gut models showed that polyethylene MPs increased potentially harmful bacteria while decreasing beneficial ones (Fournier et al., 2023). These findings highlight the potential risks of MP/NP exposure to intestinal health and emphasize the need for further research using advanced models like organoids and organ-on-a-chip systems (Bredeck et al., 2022; Yuan and Liu, 2023).

In liver, MPs can accumulate, affecting hepatocyte function and potentially contributing to liver fibrosis or other metabolic disorders.

Recent studies have investigated the impact of polystyrene microplastics (PS-MPs) on liver cell lines, revealing dose-dependent toxicity, mitochondrial dysfunction, and modification of the cellular redox state. PS-MPs and PS-NPs induce hepatotoxicity in various cell lines, including HepG2, L02, and liver organoids (Cheng et al., 2021; Guo et al., 2024; Peng et al., 2023). These particles cause mitochondrial damage, decrease ATP production, and disrupt lipid metabolism (Cheng et al., 2021; Li et al., 2023a). The key mechanism of toxicity is due to the increased production of reactive oxygen species (ROS) and the alteration of antioxidant enzyme activity. (Banerjee et al., 2022; Goodman et al., 2022; Zou et al., 2023). Particle size and surface functionalization influence cellular uptake and toxicity, with smaller and aminated particles generally causing more severe effects (Banerjee et al., 2022; He et al., 2020). These findings highlight the potential risks of PS-MPs and PS-NPs to human liver health and emphasize the need for further research on their long-term impacts.

Recent studies have shown that co-exposure to MPs and other environmental pollutants can have additive or synergistic effects on liver toxicity. Combined exposure to polystyrene MPs and benzo [a]pyrene in rats led to increased oxidative stress and liver damage (Li S. et al., 2024). Similarly, co-exposure to MPs and cadmium in mice promoted liver fibrosis through the ATP-P2X7 pathway (Sun J. et al., 2023). In zebrafish, MPs combined with cypermethrin enhanced disturbances in hepatic phospholipid metabolism and altered gut microbiota (Xu H. et al., 2024). MPs also influenced the adsorption and accumulation of triclosan in zebrafish liver, with polypropylene MPs showing the highest impact (Sheng et al., 2021). In diabetic mice, MPs disrupted glucolipid metabolism and promoted liver fibrosis (Li et al., 2023c). These findings highlight the importance of considering the combined effects of MPs and other pollutants in assessing liver toxicity risks.

Recent studies have shown that micro- and NPs can accumulate in the liver, causing significant hepatotoxicity through different mechanisms. Exposure to these particles can lead to calcium overload and subsequent hepatocyte apoptosis (Li et al., 2021a). Mitochondrial dysfunction is a key factor in this process, with particles altering membrane potential and bioenergetics (Dal Yöntem and Aydoğan Ahbab, 2024; Lin et al., 2022). The size of particles influences the type of cell death, with smaller particles primarily inducing necroptosis and larger ones causing apoptosis (Wang et al., 2023). Additionally, MPs can activate pyroptosis and ferroptosis pathways (Mu Y et al., 2022), and induce liver fibrosis through the cGAS/STING pathway (Shen et al., 2022). These effects are observed in both freshwater and seawater fish, with changes in liver function indicators (Sun Y. et al., 2024). The accumulation of micro- and NPs in the liver poses a significant threat to human and animal health (Yin et al., 2022). Further research is needed to fully understand the long-term effects. These hepatotoxic mechanisms are further corroborated by recent evidence on polyvinyl chloride microplastics (Chen Y et al., 2025) and on nickel-induced hepato-intestinal injury (Yuan S et al., 2025).

Inhalation or ingestion of MPs can lead to cellular toxicity, inflammation, and disruption of normal cellular processes. Recent studies have shown that micro- and NPs can induce the production of oxidative molecules, senescence, and apoptosis in human lung epithelial cells.

The lungs, being a primary site for airborne MPs, show evidence of tissue damage and immune response activation, which may predispose individuals to respiratory diseases.

The implications of these findings are profound, as they suggest that the widespread presence of plastic pollutants in the environment can directly impact human health.

Polystyrene MPs were found to cause alteration of oxidative balance and senescence in A549 cells (Milillo C et al., 2024), while polyvinyl chloride MPs induced senescence through ROS signaling in both A549 and BEAS-2B cells (Jin et al., 2024). Multi-walled carbon nanotubes and metal oxide nanoparticles also triggered oxidative state and apoptosis in A549 cells (Srivastava et al., 2011). Similarly, cerium oxide nanoparticles caused ROS increase and apoptosis in BEAS-2B cells (Park et al., 2008). PM2.5 exposure led to oxidative stress and autophagy in A549 cells (Deng et al., 2013). The toxicity of nanoparticles was found to be dependent on their surface charge, with highly positive charges generally inducing greater cytotoxicity (Schlinkert et al., 2015). These findings highlight the potential respiratory health risks associated with exposure to various micro- and nanoparticles.

Research on nanoparticle (NP) toxicity and uptake in the lungs reveals that surface charge and size significantly influence their biological effects. Positively charged and larger NPs tend to be more readily internalized by cells, particularly non-phagocytic ones, through various endocytotic pathways (Chou et al., 2017; Hu and Palić, 2020). This increased uptake can lead to higher cellular toxicity, primarily through reactive oxygen species generation (Chou et al., 2017; Inoue et al., 2021). Surface charge also affects lung inflammation, with more positively charged particles generally causing greater inflammatory responses (Kim et al., 2015; Warheit et al., 2006). However, surface modifications like PEGylation can enhance pulmonary retention of microparticles (Kutscher et al., 2010). In the context of vaccine delivery, cationic NPs show superior immune responses due to preferential uptake by dendritic cells and increased chemokine production (Fromen et al., 2016). These findings highlight the importance of considering surface properties when designing NPs for biomedical applications (Fröhlich, 2012).

Recent research has highlighted the role of ferroptosis, an iron-dependent form of regulated cell death, in various lung diseases. Ferroptosis is characterized by iron accumulationand lipid peroxidation. (Ma et al., 2021; Tabnak et al., 2021). It is involved in acute lung injury, chronic obstructive pulmonary disease, pulmonary fibrosis, and lung cancer (Li et al., 2021a; Xu et al., 2021). Key regulators of ferroptosis include ACSL4, NRF2, and p53 (Pan et al., 2022; Yu and Sun, 2023). The process is tightly controlled by iron, lipid, and amino acid metabolism, as well as multiple signaling pathways (Yu et al., 2021). Oxidants from cigarette smoke and asbestos fibers activate mitogen-activated protein kinase (MAPK) cascades in lung epithelial cells, leading to AP-1-dependent gene transcription involved in cell proliferation, death, and inflammation (Mossman et al., 2006). Understanding these mechanisms may provide new therapeutic targets for lung diseases (Pan et al., 2022; Xu et al., 2021).

The polystyrene microplastics (PS-MPs) have been shown to induce various detrimental effects on kidney cells. Studies have demonstrated that PS-MPs can mitochondria and endoplasmic reticulum (ER) stress, and apoptosis in human kidney cell lines such as HK-2 and HEK293 (Chen et al., 2022; Wang Y-L. et al., 2021). PS-MPs exposure leads to increased reactive oxygen species (ROS) production, altered gene expression of antioxidant enzymes, and impaired cellular proliferation (Goodman et al., 2022). Furthermore, PS-MPs can trigger inflammation, autophagy, and compromise kidney barrier integrity (Chen et al., 2022). The nephrotoxic effects of PS-MPs are exacerbated when combined with a high-fat diet, potentially promoting a profibrotic and pro-tumorigenic microenvironment (Xu W. et al., 2024). These effects involve complex interactions between ER and mitochondria, as well as the activation of various signaling pathways (Safiedeen et al., 2017). The accumulation of PS-MPs in kidney tissue suggests that long-term exposure may pose a significant risk to kidney health (Wang Y-L. et al., 2021).

Recent studies have highlighted the size-dependent toxicity and targeting potential of nanoparticles (NPs) in kidney cells. Smaller NPs generally induce greater cellular toxicity and oxidative stress (Enea et al., 2020; Passagne et al., 2012; Béatrice et al., 2008; Pujalté et al., 2011). This toxicity is attributed to increased production of reactive oxygen species free radicals and mitochondrial dysfunction (Enea et al., 2020). However, NP size also influences kidney targeting and clearance mechanisms. NPs around 75 nm can target the kidney mesangium (Choi et al., 2011), while those between 350–400 nm show broad kidney distribution (Yap et al., 2018). Factors such as charge, shape, and material density also affect renal retention (Huang Y. et al., 2021). The balance between toxic and medicinal effects depends on NP size and dose, with renal clearance modulating biological responses (Xuan et al., 2023). These findings provide crucial insights for designing kidney-targeted nanomedicines and assessing potential nephrotoxicity of nanomaterials.

Recent studies have shown that MPs can accumulate in various human organs, including the kidneys, potentially causing adverse effects (La Porta et al., 2023). Exposure to MPs at environmentally relevant doses can lead to abnormalities in renal structure and filtration function in mice (Shen et al., 2023). *In vitro* studies using human kidney cells have demonstrated that MPs can induce inflammation and alterations in protein expression (Beltrame et al., 2023; Cervello et al., 2023). MPs have been shown to cause mitochondrial dysfunction, endoplasmic reticulum stress, and autophagy in kidney cells (Wang Y-L. et al., 2021). Even at realistic environmental concentrations, polystyrene MPs can impair kidney barrier integrity and increase the risk of acute kidney injury (Chen et al., 2022). While more research is needed, these findings suggest that long-term exposure to MPs may be a risk factor for kidney disease (de Oliveira et al., 2024). Interestingly, 4-phenylbutyrate has shown potential in protecting against certain forms of acute kidney injury (Carlisle et al., 2014).

MPs pose potential risks to kidney health through various mechanisms. Studies have confirmed that MPs can accumulate in the kidneys and induce inflammation and cell apoptosis, leading to kidney injury and fibrosis (Shen et al., 2023; Xiong et al., 2023). Chronic exposure to MPs can alter gene expression related to immune response and circadian rhythm (Xiong et al., 2023), and affect oxidative phosphorylation (Shen et al., 2023). Furthermore, MPs can exacerbate cadmium-induced kidney damage by enhancing oxidative stress, autophagy, apoptosis, and fibrosis (Zou et al., 2022). While direct evidence of MPs in human kidneys is limited, their presence in various human tissues and fluids suggests potential kidney exposure (La Porta et al., 2023)). These findings highlight the need for further research on the long-term effects of MP exposure on kidney health (de Oliveira et al., 2024). These experimental findings are corroborated by *in vivo* evidence of MP-induced renal toxicity (Ijaz et al., 2024).

MPs and NPs can accumulate in ovaries and affect reproductive function in both animals and humans. Studies show that MPs and NPs can inhibit proliferation, induce apoptosis processes in granulosa cells (Fu et al., 2014; Huang W et al., 2024; Zeng et al., 2023). These effects are mediated through various pathways, including p53 signaling (Lu C et al., 2023). MPs and NPs can also disrupt steroidogenesis, reduce fertility, and cause developmental toxicity (Fu et al., 2014; Iti and Sabbir, 2022). *In vitro* studies using human granulosa cell lines like KGN and COV434 have demonstrated that MPs and NPs can enter these cells, alter their energy metabolism, and affect antioxidant responses (Bianchi et al., 2024). The reproductive toxicity of MPs and NPs is similar to that of other environmental pollutants like cadmium, which also induces apoptosis in granulosa cells through mitochondrial dysfunction (Xu et al., 2021). Consistent with this, systemic nanomaterial toxicity affecting reproductive organs has also been reported *in vivo* (Abduallah et al., 2025).

Recent studies suggest that microplastic exposure may exacerbate metastatic activity in breast cancer cells. Park J. H. et al. (2023) found that polypropylene MPs enhanced metastasis-related gene expression and cytokine secretion in breast cancer cells. Similarly, Palacios-Arreola et al. (Palacios-Arreola et al., 2021) reported that BPA, a microplastic component, increased lung metastasis in mice. Roje et al. (Roje et al., 2019) observed synergistic effects between plastic nanoparticles and parabens on breast cancer cell proliferation. Ansari et al. (Ansari et al., 2022) demonstrated that repeated BPA exposure induced metastatic aggression in breast cancer cells. Kannan and Vimalkumar (Kannan and Vimalkumar 2021) reviewed evidence linking microplastic exposure to various health effects, including potential obesogenic properties. Ramsperger et al. (Ramsperger et al., 2020) showed that environmentally exposed MPs were more readily internalized by cells. These findings, along with studies on exosomes (Mao and Jin, 2019) and hypoxia-induced microvesicles (Wang et al., 2014), highlight the complex interactions between MPs and breast cancer metastasis.

MPs have been detected in various human tissues, including semen and testes, raising concerns about their potential impact on male fertility (Li N. et al., 2024; Montano et al., 2023; Zhao et al., 2023). Studies have found MPs in semen samples from both polluted areas and the general population, with polystyrene, polyethylene, and polyvinyl chloride being the most prevalent types (Li N. et al., 2024; Montano et al., 2023). MPs exposure has been associated with alterations in sperm motility and morphology (Li N. et al., 2024; Zhang C. et al., 2022).

The presence of MPs in human reproductive tissues suggests potential routes of exposure through ingestion, inhalation, and subsequent accumulation in the epididymis and seminal vesicles. MPs are linked to negative effects on human health; animal studies and human data indicate potential risks, caused by inflammation and reproductive toxicity.

Renal cells are susceptible to the accumulation of MPs, where they can induce inflammatory responses and interfere with kidney function. In terms of reproductive health, NPs have been found to affect gamete function, disrupt hormonal regulation, and lead to alterations in the development of embryos. Gastric cells also show evidence of altered function upon exposure to MPs, with potential implications for digestive health and absorption efficiency.

The persistent exposure to MPs, whether through contaminated food, water, or air, poses a significant public health risk, underscoring the urgency of reducing plastic pollution and advancing research into the long-term effects of MPs exposure (Ferrari et al., 2024; Kyu et al., 2022; Mensah et al., 2023; Tran et al., 2022)

Oxidative stress and inflammatory responses are identified as key events in the adverse outcome pathway (AOP) framework for MP/NP-induced digestive toxicity (Ding et al., 2023; Hu and Palić, 2020), highlighting the need for further research to fully understand the public health implications.

## Conclusion

In summary, the evidence synthesized in this systematic review indicates that micro- and nanoplastics can induce apoptosis-related toxicity across a broad range of *in vitro* organ-specific models. Despite differences among target tissues, recurrent molecular mechanisms include oxidative stress, mitochondrial dysfunction, endoplasmic reticulum stress, calcium imbalance, inflammatory signaling, and activation of apoptosis-related pathways. These findings support the relevance of apoptosis as a central toxicological node, while also emphasizing the need for standardized methods and more realistic exposure scenarios before direct implications for human risk assessment can be established.

MPs and NPs are ubiquitous environmental pollutants that can enter unknowingly into the human body through ingestion, inhalation, and dermal contact (Sangkham et al., 2022; Zhu J. et al., 2024). These particles can accumulate in various organs and tissues, potentially causing adverse health effects (Fontes et al., 2024). Several studies have shown that MPs and NPs can induce oxidative stress, inflammation, DNA damage, and disrupt cellular processes (Das, 2023; González-Acedo et al., 2021) (Figure 3). They may also negatively affect the digestive, respiratory, reproductive, and nervous systems (Ali et al., 2024). The toxicity of MPs and NPs is associated with their ability to trigger some signaling pathways, including p53, MAPK, and Nrf2, which alter the physiological cellular homeostasis (Khan and Jia, 2023). Additionally, these particles can carry toxic chemicals and pathogens, potentially exacerbating their harmful effects (Kumar et al., 2022). While current research highlights the potential risks of MPs and NPs, further studies are needed to fully understand their long-term impacts on human health (Ali et al., 2024). Discussion on the potential long-term health implications of widespread microplastic contamination in the environment is critical, as chronic exposure could lead to cumulative health risks, including the development of chronic diseases and immune system dysfunction. Understanding the cellular mechanisms behind these effects, especially in organs like the liver, lungs, and gut, is essential for assessing the long-term consequences. Given the persistence of MPs in the environment and their widespread distribution, there is an urgent need for research to evaluate the cumulative effects of chronic exposure. Moreover, a call for developing regulatory frameworks is necessary to limit exposure and mitigate the risks associated with microplastic pollution, ensuring a more sustainable and healthier environment for future generations.
